# Practical considerations for quantitative clinical SPECT/CT imaging of alpha particle emitting radioisotopes

**DOI:** 10.7150/thno.63860

**Published:** 2021-09-27

**Authors:** Nadia Benabdallah, William Scheve, Nicholas Dunn, Delynn Silvestros, Paul Schelker, Diane Abou, Uday Jammalamadaka, Richard Laforest, Zekun Li, Jonathan Liu, David H. Ballard, Nichole M. Maughan, Hiram Gay, Brian C. Baumann, Robert F. Hobbs, Buck Rogers, Amir Iravani, Abhinav K. Jha, Farrokh Dehdashti, Daniel L. J. Thorek

**Affiliations:** 1 Department of Radiology, Washington University School of Medicine, St. Louis, Missouri.; 2 Program in Quantitative Molecular Therapeutics, Washington University School of Medicine, St. Louis, Missouri.; 3 Barnes-Jewish Hospital, St. Louis, Missouri.; 4 Department of Biomedical Engineering, Washington University, St. Louis, Missouri.; 5 Department of Radiation Oncology, Washington University School of Medicine, St. Louis, Missouri.; 6 Radiation Oncology and Molecular Radiation Sciences, Johns Hopkins University School of Medicine, Baltimore, Maryland.; 7 Oncologic Imaging Program, Alvin J. Siteman Cancer Center, Washington University School of Medicine, St. Louis, Missouri.

**Keywords:** radiopharmaceutical therapies, quantitative SPECT/CT, Radium-223, Actinium-225, Thorium-227

## Abstract

**Rationale:** Alpha particle emitting radiopharmaceuticals are generating considerable interest for the treatment of disseminated metastatic disease. Molecular imaging of the distribution of these agents is critical to safely and effectively maximize the clinical potential of this emerging drug class. The present studies aim to investigate the feasibility and limitations of quantitative SPECT for ^223^Ra, ^225^Ac and ^227^Th.

**Methods:** Three state-of-the-art SPECT/CT systems were investigated: the GE Discovery NM/CT 670, the GE Optima NM/CT 640, and the Siemens Symbia T6. A series of phantoms, including the NEMA IEC Body phantom, were used to compare and calibrate each camera. Additionally, anthropomorphic physical tumor and vertebrae phantoms were developed and imaged to evaluate the quantitative imaging protocol.

**Results:** This work describes and validates a methodology to calibrate each clinical system. The efficiency of each gamma camera was analyzed and compared. Using the calibration factors obtained with the NEMA phantom, we were able to quantify the activity in 3D-printed tissue phantoms with an error of 2.1%, 3.5% and 11.8% for ^223^Ra, ^225^Ac, and ^227^Th, respectively.

**Conclusion:** The present study validates that quantitative SPECT/CT imaging of ^223^Ra, ^225^Ac, and ^227^Th is achievable but that careful considerations for camera configuration are required. These results will aid in future implementation of SPECT-based patient studies and will help to identify the limiting factors for accurate image-based quantification with alpha particle emitting radionuclides.

## Introduction

The application of radiation to treat primary and oligometastatic disease is a pillar of cancer therapy, demonstrating efficacy in a wide range of cancer types. However, in the metastatic setting, image-guided application of conventional external beam irradiation is generally limited to palliative use. This is because the inherent challenges in safely and effectively applying sufficient dose to multiple sites throughout the body, including occult micrometastases, make this approach infeasible. Systemic administration of radiopharmaceutical therapies (RPT) have the potential to deliver ablative doses to myriad lesions, and this modality has a long history of established clinical practice 1. Here, pretherapeutic imaging and pharmacokinetic information of RPT distribution are important to measure absorbed doses at both sites of disease and to avoid off-target normal organ toxicities.

There has been a growing interest in application of alpha particle (⍺)-emitting RPT for metastatic disease. Alpha particles are charged helium nuclei that deposit 5-10 MeV of energy in the range of only 50-100 of microns, resulting in potent cytotoxicity. This produces complex DNA damage to both cancer cells and normal tissues relative to conventional radiotherapies. The biological impact and physical characteristics of these emissions make the quantitative imaging of the activity distribution essential in order to optimize administered activities, modify treatment plans, monitor accumulation, and mitigate toxicity. There are several ⍺-emitting radioisotopes with relevant biomedical properties including ^212^Pb (parent of ⍺-emitting ^212^Bi; t_1/2_ = 10.6 h) and ^211^At (t_1/2_ = 7.2 h). Among the most translationally relevant, and the focus of this work, are the longer-lived ^223^Ra (t_1/2_ = 11.4 days), which is approved for use in treatment of metastatic castrate-resistant prostate cancer (mCRPC), its parent, ^227^Th (t_1/2_ = 18.7 days), and ^225^Ac (t_1/2_ = 10 days).

Despite the long-standing interest, there are few reports of quantitative imaging methods for assessment of ⍺-RPT. Indeed, the combination of a complex photon emission spectrum and the very low activities administered to patients makes imaging of ⍺-emitting radiopharmaceuticals challenging because of the low number of detectable photons. There is also a need to compensate for the scatter and septal penetration that constitutes a substantial part of the acquired signal.

The efficacy of ^223^Ra in mCRPC patients has been demonstrated in multiple studies 2-11 and this radionuclide has three principal gamma emissions at 81, 154, and 270 keV 12. Multiple reports have shown that it is possible to acquire coarse imaging of ^223^Ra using planar methods 13-16, primarily using an energy window centered on the 81 keV gamma emission. Such gamma camera planar imaging can be used to assess biodistribution and to calculate normal organ dosimetry in patients treated with ^223^Ra-dichloride 14, 17-22, according to the Medical Internal Radiation Dose (MIRD) formalism. Recently, several studies have shown the feasibility of quantitative single-photon emission computed tomography (SPECT) 23-25 using physical-phantom measurements. For example, Gustafson *et al.* 24 showed that quantification of a 26.4 mL sphere in a NEMA IEC Body phantom was achievable with errors ranging from -25% to -34% with a GE Infinia Hawkeye SPECT/CT gamma-camera (GE, Fairfield, CT, USA) equipped with a medium-energy collimator.

^225^Ac is currently used for antibody- and peptide-labeled therapies under pre- and clinical evaluation. In particular, there has been great interest in ^225^Ac-PSMA-617 in several investigational studies for patients with prostate cancer 26-28. For patients with advanced disease, treatment activities of 100 kBq/kg of ^225^Ac-PSMA-617 per cycle repeated every 8 weeks have been administered 26, 27. ^225^Ac has two principal gamma emissions at 218 keV and 440 keV 12 from its daughters ^221^Fr and ^213^Bi respectively, along with bremsstrahlung X-rays from ^209^Pb. Kratochwil *et al.* 28 have reported planar imaging of patients treated with ^225^Ac-PSMA-617 using energy windows centered around the two gamma emission lines previously noted. In addition, Usmani *et al.* recently demonstrated that addition of a 78 keV photopeak provided improved quality of images with a clearer distinction of lesions 29. To the best of our knowledge, a single report of quantitative ^225^Ac imaging (for PSMA-targeted therapy) has been evaluated 30. However, the ^213^Bi daughter has been investigated for tomographic imaging with ^213^Bi-DOTATOC. These have shown promising initial results 31.

Finally,^ 227^Th is also the subject of several early-phase clinical trials using antibody targeted vehicles, such as an alternative PSMA construct in mCRPC patients (NCT03724747) 32, 33. ^227^Th has one principal gamma emission at 236 keV 12, and is the parent of ^223^Ra; thus, its gamma emissions can also be used for imaging. According to the work of Murray *et al.* 34, planar imaging of ^227^Th is achievable using multiple energy windows, and this approach may provide quantitative value. Quantification of a 3 cm diameter sphere in a water-filled cylinder phantom ^227^Th filled phantom by SPECT provided values within an error of -12.4 ± 4.9%, using a Symbia Intevo (Siemens) gamma camera equipped with a medium energy-low penetration collimator 35.

These initial efforts for planar and tomographic imaging of ^223^Ra, ^225^Ac, and ^227^Th are encouraging. They motivate further characterization of the properties of SPECT-based activity quantification for ⍺-RPT to allow for its use in patient studies to acquire reliable information about their accumulation in both sites of disease and at-risk organs. In general, SPECT is considered superior to planar imaging for activity quantification 36 with the primary advantage of eliminating organ activity overlap and enabling more accurate attenuation and scatter correction.

The present studies investigate the feasibility and limitations of quantitative SPECT for ^223^Ra, ^225^Ac, and ^227^Th. A series of phantom measurements were performed on three state-of-the-art SPECT/CT systems: GE Discovery NM/CT 670, GE Optima NM/CT 640 and Siemens Symbia T6. This work describes and validates a methodology to calibrate each camera. The efficiency of each gamma camera is analyzed and compared, with a primary focus on the estimation of radioactivity concentration in lesions. The long-term goal is to characterize these properties to allow for future SPECT/CT-based patients studies and to identify the limiting factors for accurate image-based quantification with these potent radiopharmaceuticals.

## Methods

### Radioisotopes

In this work, three ⍺-emitters have been studied: ^223^Ra, ^225^Ac, and ^227^Th. Figure S1 illustrates each radionuclide's decay scheme and Table 1 summarizes the principal photon emissions. For imaging evaluation, five phantoms were used: the standardized NEMA IEC Body phantom, two custom detection-limit phantoms, a tumor phantom, and a vertebrae phantom. ^225^Ac and ^227^Th were supplied as dry nitrates from the Department of Energy (Oak Ridge National Laboratory) from ^228^Th and ^227^Ac-generator systems, respectively.^ 227^Th samples for spectroscopic and tomographic evaluation were chemically purified at maximum of one day prior to their use. Different sources of ^223^Ra solutions were used for these studies. Xofigo (^223^RaCl_2_) was used for energy spectrum and sensitivity measurements. For the NEMA phantom and the high activity limit of detection phantom, ^223^Ra was generated in our laboratory from trace ^227^Ac, as previously reported 37. For the low activity limit of detection phantom and the vertebrae phantom, ^223^Ra was produced in our laboratory from ^227^Th. All samples used for measurement were quality controlled by high resolution gamma spectroscopy in order to verify their identical radiological properties (for ^223^Ra sources), and purity. A 1.5 mL conical polypropylene vial was filled with 37 kBq of each radioisotope were measured for 10 min on a high purity germanium (HPGe) system (GEM-50195-S; Ortec). Samples were placed directly on a high purity aluminum endcap and the system was enclosed in a 10 cm lead shield (HPLBS1, Ametek). Spectral acquisitions were acquired and analyzed by Gamma-Vision Software (version 8.0, Ametek).

### Activity measurements

All sample activities were carefully measured with the same ionization chamber radionuclide dose calibrator (CRC-15; Capintec). The dial settings used for ^223^Ra, ^225^Ac, and ^227^Th are 236, 72, and 91, respectively. A National Institutes of Standards and Technology traceable source was used to empirically determine the dose calibrator value for ^223^Ra, as previously described 36. A National Physics Laboratory (UK) traceable source, in preparation for investigation clinical treatment studies with antibody conjugates, was used to determine the dose calibrator value for ^227^Th, and verified by HPGe measurements.

### SPECT/CT camera characteristics

To evaluate vendor, collimator, and device-specific effects on low activity ⍺-emitter SPECT acquisition, we evaluated three dual-head SPECT/CT cameras: a Discovery 670 and an Optima 640 SPECT/CT from GE and a Symbia T6 from Siemens. All the cameras were equipped with their specific set of collimators: MEGP (medium energy general purpose) for GE or MELP (medium energy low-penetration) for Siemens and HEGP (high-energy general purpose) for both vendors. Enclosed in Table 2 and Table 3 are the camera system and collimator characteristics of each unit, respectively.

### Energy spectrum and sensitivity comparison

To set the energy windows for quantitative imaging, the energy spectrum of each radioisotope was measured on the Discovery 670 (GE) and Symbia T6 (Siemens) with both collimators. Since the gamma camera components from the Discovery 670 and the Optima 640 are identical, the energy spectrum was measured only on the Discovery 670. The source was contained in a 5 mL glass vial and placed in the center of the field of view between the two gamma camera heads at a point 17 cm from both heads. The activity in the source was 932 kBq, 2242 kBq, and 419 kBq for ^223^Ra, ^225^Ac, and ^227^Th, respectively.

The Symbia T6 offers the possibility of directly measuring the energy spectrum without acquiring an image. For the measurement on the Discovery 670, the energy spectrum was assessed from planar images of each source, acquired using energy windows covering the range from 57 keV to 475 keV, totaling 59 energy windows for ^223^Ra and ^227^Th and 43 energy windows for ^225^Ac. Each acquisition was acquired for a duration of 5 min. The emission energy windows were selected to provide coverage of the relevant photopeaks specific for each radioisotope, and a corresponding scatter energy window was estimated on adjacent lower energies.

In order to compare the performance of each system, the sensitivity was measured for each radioisotope and collimator. The same geometry and sources, as described above, were used. Planar images of each source, and an acquisition without any source for background subtraction, were recorded using the emission windows determined from the energy spectrum for a duration of 10 min. The sensitivity, denoted by S, was calculated as follows:


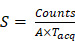

(1)

where Counts denotes the total counts measured in a region of interest (ROI) with the same area as the vial and centered around it, after background subtraction; T_acq_ is the acquisition duration (s), and A denotes the nominal activity in the vial at the time of acquisition (MBq).

### Quantitative phantom study

A NEMA IEC Body phantom (Data Spectrum) was used to investigate quantitative SPECT/CT imaging characteristics. The NEMA phantom contains 6 spheres of different diameters: 3.7, 2.8, 2.2, 1.7, 1.3, and 1.0 cm. Each sphere was filled with the same activity concentration of the radioisotope: 40 kBq/mL for ^223^Ra, 35 kBq/mL for ^225^Ac, and 40 kBq/mL for ^227^Th, with the main cavity of the phantom body filled with non-radioactive water.

The phantom was placed in the center of the field of view and SPECT/CT acquisitions were performed with both MEGP/MELP and HEGP collimators on each system with energy windows determined from energy spectrum evaluation. The body-contour acquisition orbit was utilized and 30 s acquisitions per projection were acquired for every 6°, over a 360° orbit.

The images acquired on the GE cameras were reconstructed on the Xeleris software with the following parameters: 2D-OSEM algorithm, 2 iterations, 10 subsets and Butterworth filter (f_c_ = 0.48, p = 10). The projections were corrected for scatter using the corresponding scatter windows and the dual-energy window (DEW) scatter-compensation method 38 and attenuation using the CT-based attenuation map. Indeed, during a SPECT/CT acquisition, one image was generated for each emission energy window and each scatter energy window. The software also acquired CT-based attenuation maps with attenuation coefficients corresponding to each emission energy window. Finally, the reconstructed images were summed to evaluate combinations of energy windows.

The acquired data on the Symbia T6 were reconstructed on the Esoft workstation (Siemens) with the following parameters: 3D OSEM algorithm, 16 iterations, 4 subsets and Gauss filter (FWHM = 9 mm). Attenuation correction was performed from the CT using manufacturer's default parameters. They were not corrected for scatter, as this software does not enable scatter correction for multiple energy window acquisitions. Similar to the Xeleris software, the Esoft workstation reconstructs each energy window individually. Table 2 summarizes the acquisition and reconstruction parameters used for the evaluation of quantitative imaging of the phantom.

The reconstructed images were analyzed using HERMES (v2.7.2, Hermes Medical Solutions). Volumes of interest (VOIs) defined on the CT images were applied to the SPECT data: 6 spheres of the same dimensions of the spherical inserts of the NEMA phantom. The total number of counts was obtained for each sphere.

The calibration factor CF (counts/s/MBq) was calculated for each sphere 39:


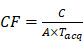

(2)

where C denotes the total reconstructed counts within the target volume of interest, A is the activity present in the sphere at the time of acquisition and T_acq_ denotes the duration of the acquisition.

**Figure 1:**
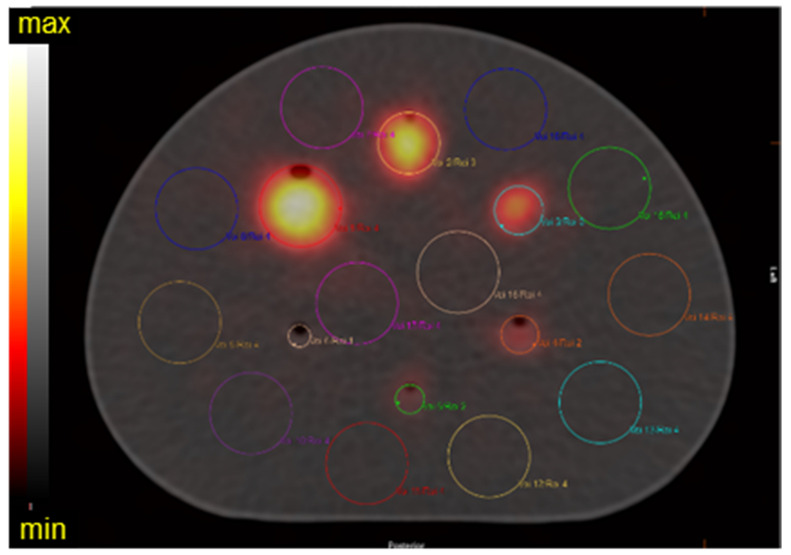
Target slice of the SPECT/CT fusion of the NEMA phantom with activity-filled spheres. Location of VOIs for analysis are indicated, along with the background VOIs (corresponding to the largest sphere volume; 26.5 mL). This representative reconstruction of the phantom is of ^223^Ra acquired on the Discovery 670 with a MEGP collimator.

To compare imaging noise metrics, the central slice traversing all six spheres was selected as the target slice for analysis. A total of 36 background spherical VOIs were defined for each sphere size (3.7, 2.8, 2.2, 1.7, 1.3, and 1.0 cm diameter) by positioning 12 background VOIs on the target slice, as well as on two neighboring slices (slice positions ± 20 mm) (Figure 1). The number of counts in each VOI was reported using HERMES Hybrid Viewer and they were used to calculate the signal to noise ratio (SNR) and background variability (BV) 40. The SNR was defined as:


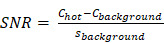

(3)

where C_hot_ denotes the number of counts detected in a hot sphere, C_background_ denotes the mean of number of counts detected in the corresponding background VOIs, and s_background_ denotes the standard deviation of the background VOIs.

At the low detected counts for αRPT, orders of magnitude reduced from conventional SPECT tracer studies (typically with ^99m^Tc), the signal generated in background following reconstruction is not negligible. BV measures the variation of VOI means over multiple VOIs. It takes into account the scatter and the ensemble noise in the reconstructed image. The BV was calculated with the following:


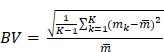

(4)

where m_k_ denotes the mean of pixels in k^th^ background VOI, 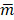 denotes the average of background VOI means over different VOIs and K denotes the number of background VOIs (K = 36).

### Limits of detection

**Figure 2:**
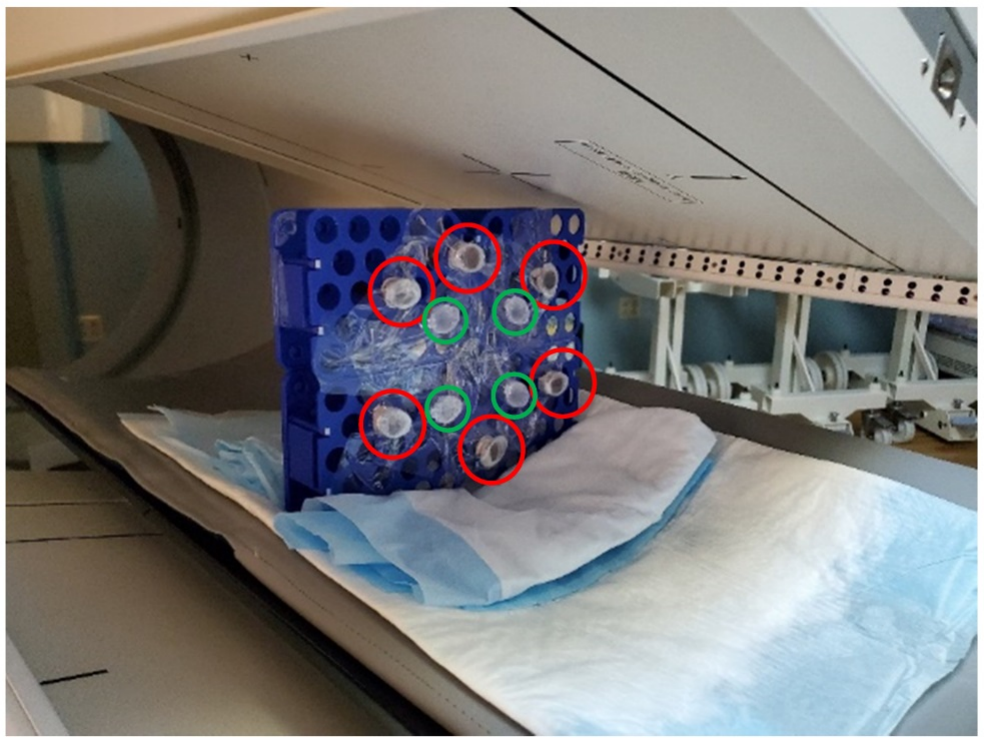
Limit of detection phantom scanned by the Optima 640 gamma-camera. The six identical conical polypropylene vials are filled with different concentrations of ^223^Ra: 74, 37, 27.7, 18.5, 9.2, and 3.7 kBq/mL. The red circles denote the vials containing the hot sources while the green circles denote the vials filled with water.

A laboratory-built phantom was created to assess the limits of detection for each system, configuration, and isotope. This phantom is comprised of six 5 mL conical polypropylene vials filled with a range of activity concentrations of each radioisotope and four 5 mL conical polypropylene vials filled with water arranged at the center (Figure 2). To cover the entire range of physiologically relevant activities, two identical phantoms covering alternate ranges of activity were evaluated. In the first (high), vials contained 74, 37, 27.7, 18.5, 9.2, and 3.7 kBq/mL of each radioisotope and in the second (low), the vials were filled with 20, 15, 5, 2, 1, and 0.5 kBq/mL. SPECT/CT acquisitions were acquired of each limit of detection range, on each gamma camera and with both collimators. The acquisition and reconstruction parameters were identical to those described above.

VOIs were defined as 10 ellipsoids surrounding each vial, applied as above. The total number of counts was obtained for each VOI and plotted in function of the concentration of the radioisotope in the vials. The limit of detection was achieved when the number of counts in the VOI around the hot vial was not above 10 times the number of counts in the background. The background is determined as the mean number of counts in the VOIs surrounding the water filled vials.

### Validation with anthropomorphic physical phantoms

In order to validate the acquisition and reconstruction protocol, a realistic tumor phantom was imaged. In collaboration with the 3D Printing Lab at Washington University, we modeled volumes representative of a realistic tumor using patient-data-derived tumor properties (size, shape) and a vertebrae based on a patient CT image. Computer-aided designs (CADs) were created in Blender V 2.82. Molds and the lid of the phantom were printed on Formlabs Form 2 3D printer with elastic resin. Two fillable 3D phantoms were printed (Figure 13 and Figure S12) 41. The printed vertebrae phantom was covered with a solution of Plaster of Paris (calcium sulfate hemihydrate) to accurately mimic bone attenuation. The volumes of the tumor and vertebrae phantoms were 12 mL and 15 mL, respectively. The choice of the tumor phantom volume was based on patient-data-derived tumor volume and on selecting a volume similar to one of the NEMA sphere for quantification purposes. The vertebrae phantom was filled with 40 kBq/mL of ^223^Ra and the tumor phantom was filled with 35 kBq/mL of ^225^Ac and ^227^Th. For each configuration, the phantom was fixed on the cylindrical insert inside the NEMA phantom, which was filled with non-radioactive water to recapitulate body scatter and attenuation features.

A SPECT/CT acquisition on the Discovery 670 gamma camera with the MEGP collimator was acquired for each phantom. The acquisition and reconstruction parameters are the same as those described above. A VOI was contoured on the CT and applied on the SPECT data and counts inside both tumor and vertebrae were determined. The activity in the tumor was estimated using the calibration factor as obtained from the experiments with the NEMA phantom and compared to the true activity.

## Results

### Radioisotopes

**Figure 3:**
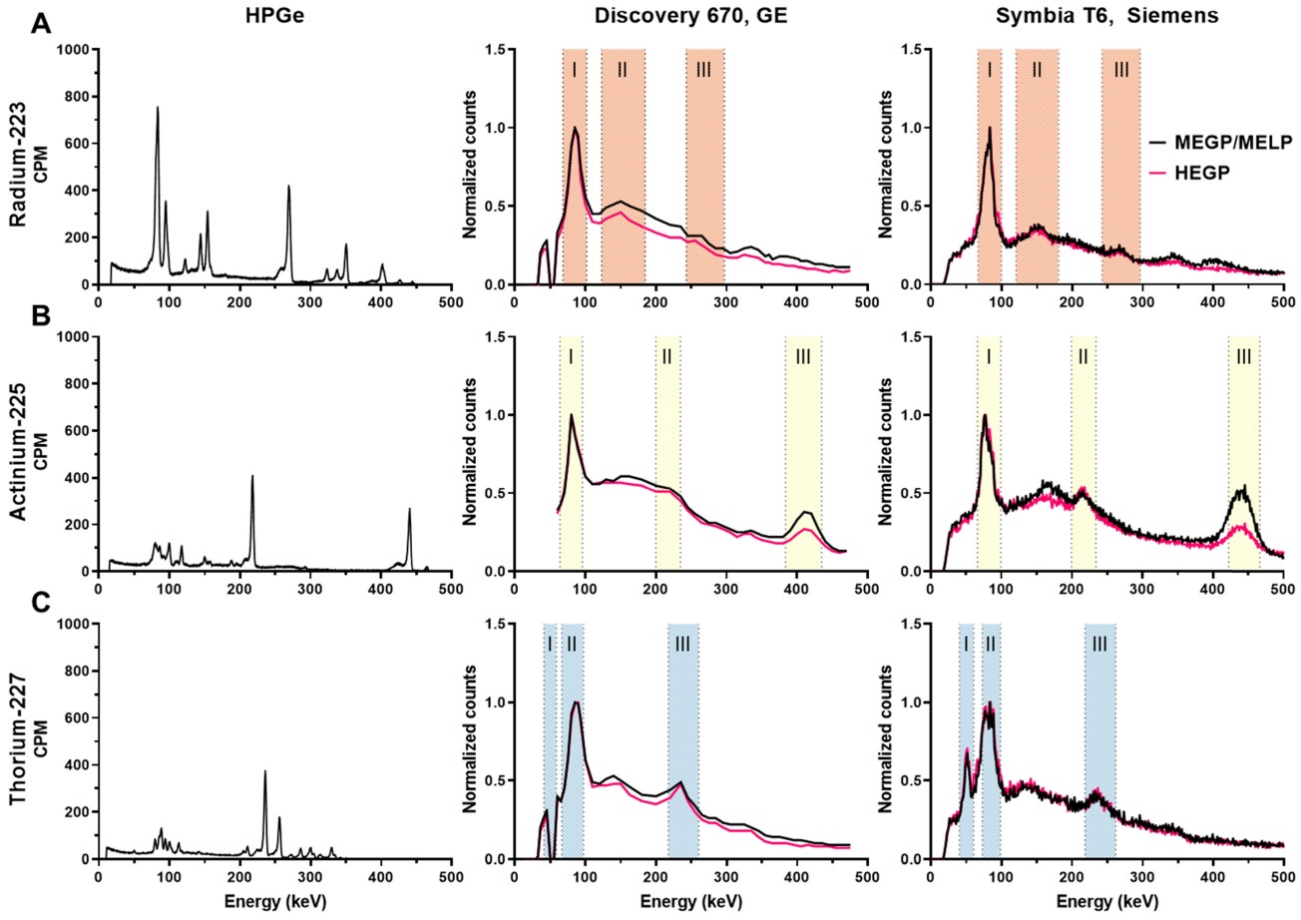
High resolution and clinical imaging system derived energy spectra of: A) ^223^Ra, B) ^225^Ac, C) ^227^Th. Left column: energy spectra measured by HPGe. Middle column: energy spectra measured on the Discovery 670 (GE) with the MEGP and HEGP collimators. Right column: energy spectra measured on the Symbia T6 (Siemens) with the MELP and HEGP collimators. The shaded areas are the chosen emission energy windows for SPECT evaluation.

Figure 3 (left panel) shows the high-resolution gamma spectra of each source of the radioisotope from measurements using the HPGe. The principal photon emissions described in Table 1 are detected. The characteristic gammas are 81.1, 83.8, 94.9, 154.2, and 269.5keV for ^223^Ra; 218.2 and 440.5 keV for ^225^Ac, and 236 keV for ^227^Th.

### Energy spectrum

The energy spectrum for each isotope measured on the Discovery 670 and the Symbia T6 are shown in Figure 3 middle and left panel, respectively. The characteristic photopeaks of each isotope were measured and defined by both gamma cameras.

For ^223^Ra, the 81.1 (15.0%), 83.8 (24.9%), and 94.9 keV (11.3%) emissions were detected in a same peak as were the 269.5 (13.7%) and 271.2 keV (10.8%) emissions because of the limited gamma camera energy resolution. The photons at 351.1 keV (12.9%) from ^211^Bi was also visible in a photopeak at 350 keV. Moreover, the Symbia T6 measured both gammas at 401.8 (6.4%) and 404.9 keV (3.8%) in one peak with the MELP collimator, contrary to the Discovery 670, which did not resolve this emission.

For ^225^Ac, two photopeaks apart from characteristic X-rays (around 80 keV) coming from the collimator were visible: the one at 218.2 keV (11.6%) from ^211^Fr and at 440.5 keV (26.1%) from ^213^Bi. We observed that while the characteristic 440 keV emission of the ^213^Bi daughter of ^225^Ac was detected appropriately at 440 keV on the Symbia T6, this emission line was identified at 410 keV on the Discovery 670. On both gamma cameras, we noted a significant down scatter peak around 170 keV.

For ^227^Th, several peaks apart from the combined peaks for characteristic X-rays (around 82 keV) were visible. For example, the 236 keV (12.3%) gammas from ^227^Th were detected. Moreover, the Symbia T6 gamma camera measured the 50.1 keV (8.0%) gammas from ^227^Th, contrary to the Discovery 670 on which we observed a decrease in signal, an aberration that may be due to a specific energy channel issue with our system. As the ^227^Th source was purified the day before the measurement, the characteristic gammas of ^223^Ra were not discernable.

The emission and scatter energy windows selected from the energy spectra measurements are summarized in Table 4 and Table 5 for the GE and the Symbia T6 Siemens SPECT/CT, respectively. Scatter windows were not used for the Symbia T6 system, as the system software does not allow the scatter correction on multiple energy windows acquisition.

**Figure 4:**
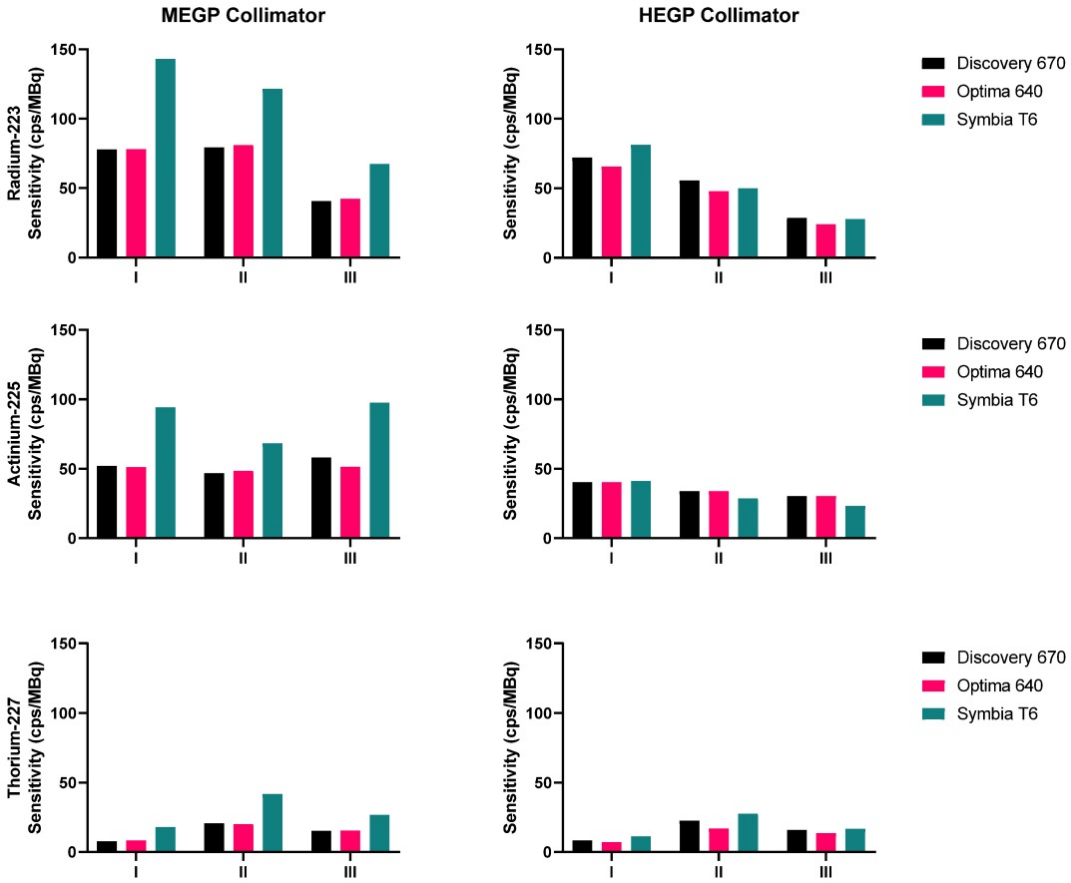
Sensitivities measured on the three gamma cameras with both collimators, for the three isotopes: ^223^Ra (top), ^225^Ac (middle), ^227^Th (bottom). The energy windows I, II and III are: 85 keV ± 20%, 154 keV ± 20%, and 270 keV ± 10% for ^223^Ra; 82 keV ± 20%, 216.8 keV ± 8%, and 444.3 keV ± 5% for ^225^Ac; 50.6 keV ± 20%, 85.7 keV ± 15%, and 240 keV ± 9% for ^227^Th.

The sensitivities measured for ^223^Ra, ^225^Ac and ^227^Th for each gamma camera configuration and each emission window are presented in Figure 4. For each isotope, the results obtained on the Symbia T6 with the MELP collimator yielded the greatest computed sensitivity. For each isotope, the sensitivity of the HEGP collimator was lower than that determined with the MEGP/MELP collimator. Measurement with ^223^Ra achieved their highest sensitivity when summing the three energy windows, which was consistent across images using the MEGP/MELP collimators for the three isotopes (Figure 3, left column).

### Quantitative phantom study

**Figure 5:**
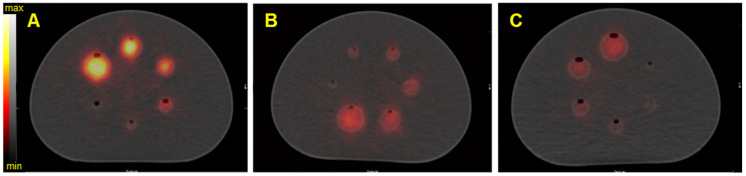
SPECT/CT fusion of the NEMA phantom acquired on the Discovery 670 (GE) with the HEGP collimator. Images are of A) Spheres filled with 40 kBq/mL of ^223^Ra; B) spheres filled with 35 kBq/mL of ^225^Ac, C) spheres filled with 40 kBq/mL of ^227^Th.

Figure 5 shows the reconstructed fused SPECT/CT images of the NEMA phantom for the three isotopes with the Discovery 670 SPECT/CT equipped with the HEGP collimator. The emission and scatter energy windows used for each isotope and each gamma camera are summarized in Table 4 and Table 5.

As expected from the sensitivity results (Figure 4), the resulting SPECT/CT images with ^223^Ra had the greatest number of counts in the reconstructed volumes. Except in the smallest sphere (20.8 kBq for ^223^Ra and ^227^Th and 18.2 kBq for ^225^Ac), all of the spheres were visible. For brevity, representative reconstructions for all additional data are presented in the Supplemental Data.

For each reconstructed image and each sphere dimension, the CF, SNR and BV were assessed.

#### Ra-223

As shown in Figure 8, on the Discovery 670 (GE), the highest SNR with the MEGP and HEGP collimators was obtained with the window I (85 keV ± 20%) and the window III (270 keV ± 10%), respectively. Compared to the MEGP collimator, the HEGP gave a higher calibration factor values: 2.5% more for the largest sphere (3.7 cm diameter). However, the BV was higher (1.3% for the largest sphere-volume compared to the MEGP collimator) and the SNR was lower. For instance, in the case of the largest sphere, the SNR was 1.4% lower with the HEGP collimator than with the MEGP collimator.

Analyzing imaging data from the Optima 640 (GE) showed that SNR for the window III was greater with the HEGP collimator than with the MEGP collimator (Figure S2). The MEGP collimator gave a higher calibration factor and SNR; for example, with the second largest sphere (2.8 cm diameter), they were 5.3% and 1.3% greater than with the HEGP collimator. However, the BV was increased: 6.1% for the second largest sphere compared to the HEGP collimator.

Similarly, using the Symbia T6 (Siemens), the HEGP collimator yielded improved SNR for the window III (Figure S3). Acquisitions with the MELP collimator provided a higher calibration factor: 6.8% greater for the largest sphere than with the HEGP collimator. However, the BV was increased: 2.0% for the largest sphere compared to the HEGP collimator. The HEGP collimator gave increased SNR: 8.3% higher for the largest sphere.

To summarize, for ^223^Ra acquisition parameters and quantitative SPECT image metrics, the Symbia T6 with the HEGP collimator has given the highest calibration factor and the lowest BV. However, except for the largest sphere volume, the acquisitions using the Discovery 670 with the HEGP collimator resulted in greater SNR.

#### Ac-225

Using the Discovery 670 with the MEGP collimator without scatter correction provided improved imaging results (Figure S4) than with correction, and these data are consistent with the energy spectrum analysis. Indeed, the calibration factor and SNR were 11.8% and 3.6% higher for the largest sphere than with scatter correction. However, the BV was 3.4% greater without scatter correction. The results were similar for the HEGP collimator (Figure S5). The calibration factor, BV and SNR were 12.9%, 0.8% and 10.9% higher for the second largest sphere compared to with scatter correction. To summarize, the calibration factor for every sphere was similar with both collimators (only 1.4% different with the MEGP collimator for the largest sphere than with the HEGP collimator, which was not significant) and a lower BV. However, the SNR was higher with the HEGP collimator: 8.8% for the largest sphere compared to the MEGP collimator.

Similarly, using the Optima 640 with MEGP collimation also demonstrated improved results without scatter correction (Figure 7 and Figure S6). The calibration factor and SNR were 13.3% and 3.6% greater for the largest sphere than with scatter correction. However, the BV increased by 2.6%. The HEGP collimator also provided better results without scatter correction (Figure 9). The calibration factor and SNR were 14.6% and 12.6% greater for the second largest sphere than with scatter correction. However, the BV was 3.0% greater. In summary, the HEGP collimator offered a similar calibration factor (1.1% for the largest sphere compared to MEGP, which was not significant) and increased SNR (5.6% for the largest sphere). Moreover, the BV was higher with the MEGP collimator: 10.6% for the largest sphere.

On the Symbia T6, the HEGP collimator offered a better calibration factor and SNR: 3.7% and 5.6% for the largest sphere (Figure S7). However, the BV was higher with the HEGP collimator: 6.6% for the largest sphere.

Overall, the Symbia T6 with the HEGP collimator gave the highest calibration factor and SNR across the systems tested for ^225^Ac. For example, in the case of the largest sphere, it was 49.7% and 9.5% more than the Discovery 670 and 49.7% and 2.5% higher than the Optima 640. However, the BV was also increased for the largest sphere obtained with the Symbia T6: 5.7% compared to the Discovery 670 and 6.7% to the Optima 640.

#### Th-227

Acquisitions using the Discovery 670 showed that the window I (50 keV ± 17.5%) and the window III (239 keV ± 9%) had similar calibration factors with the MEGP collimator (Figure S8). The window II (82 keV ± 19%) offered the highest calibration factor and the 239 keV emission window the highest SNR. The results were slightly better without scatter correction. Indeed, the calibration factor and SNR were 3.2% and 1.1% higher than with scatter correction for the largest sphere. These observations were the same for the HEGP collimator (Figure S9). The calibration factor and SNR were 3.0% and 0.8% higher than with scatter correction for the largest sphere. Consequently, the MEGP collimator offered a better calibration factor and SNR: 2.5% and 7.6% for the second largest sphere compared to the HEGP collimator. Moreover, the BV was higher with the HEGP collimator: 1.0% for the second largest sphere.

On the Optima 640 system, the calibration factors, SNR and BV were also similar for the MEGP and HEGP collimators. For both collimators, the windows I and III (50 keV and 239 keV) had similar calibration factors. The second emission window (82 keV) offered the highest calibration factor, which was consistent with the sensitivity results (Figure S10 and Figure S11). With the MEGP collimator, the calibration factor was higher without scatter correction: 2.9% for the second largest sphere. However, the SNR was higher and the BV was lower with scatter correction: 2.2% and 2.1% for the second largest sphere. With the HEGP collimator, the calibration factor and SNR were higher without scatter correction: 3.3% and 0.6% for the largest sphere. As a result, the HEGP collimator gave a higher calibration factor: 3.0% for the second largest sphere. However, the SNR was higher and the BV was lower with the MEGP collimator: 5.5% and 5.5% for the second largest sphere.

**Figure 6:**
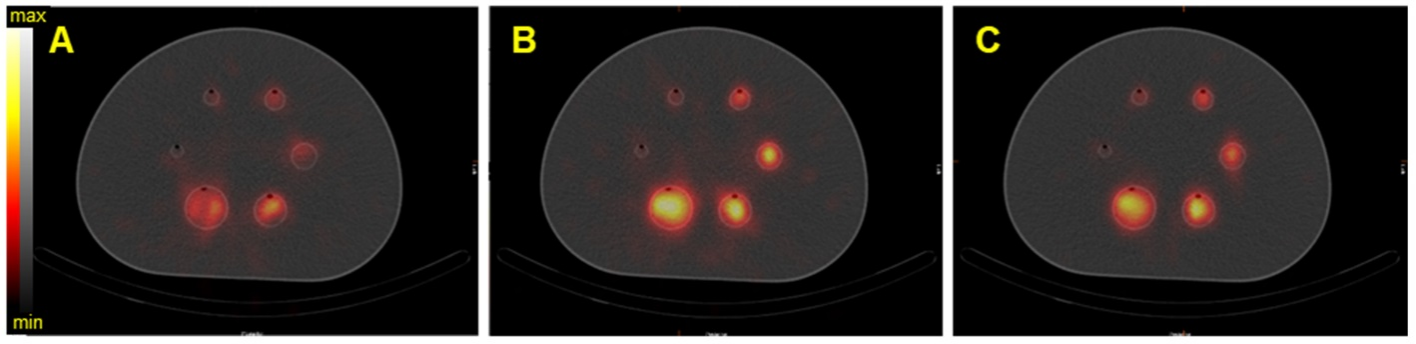
SPECT/CT fusion of the NEMA phantom acquired on the Symbia T6 (Siemens) with the MELP collimator. The phantom was filled with 40 kBq/mL of ^227^Th. A) Reconstructed image of the 50.6 keV ± 20% emission window, B) reconstructed image of the 85.7 keV ± 15% emission window, C) reconstructed image of the 240 keV ± 9% emission window.

**Figure 7:**
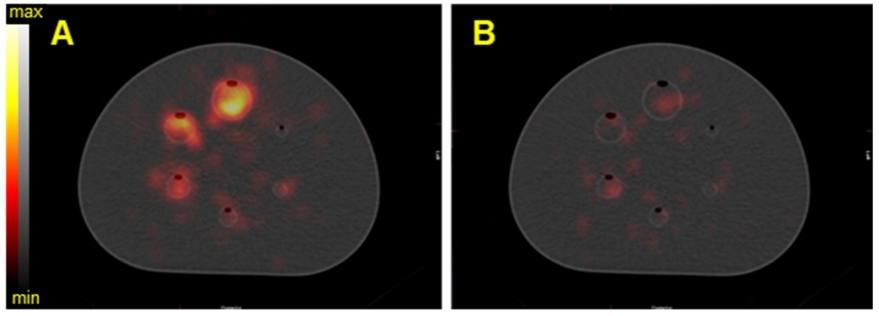
SPECT/CT fusion of the NEMA phantom acquired on the Optima 640 (GE) with the MEGP collimator. The phantom spheres were filled with 35 kBq/mL of ^225^Ac. A) Reconstructed image of the 217.5 keV ± 8% emission window without scatter correction, B) Reconstructed image of the 217.5 keV ± 8% emission window with scatter correction.

**Figure 8:**
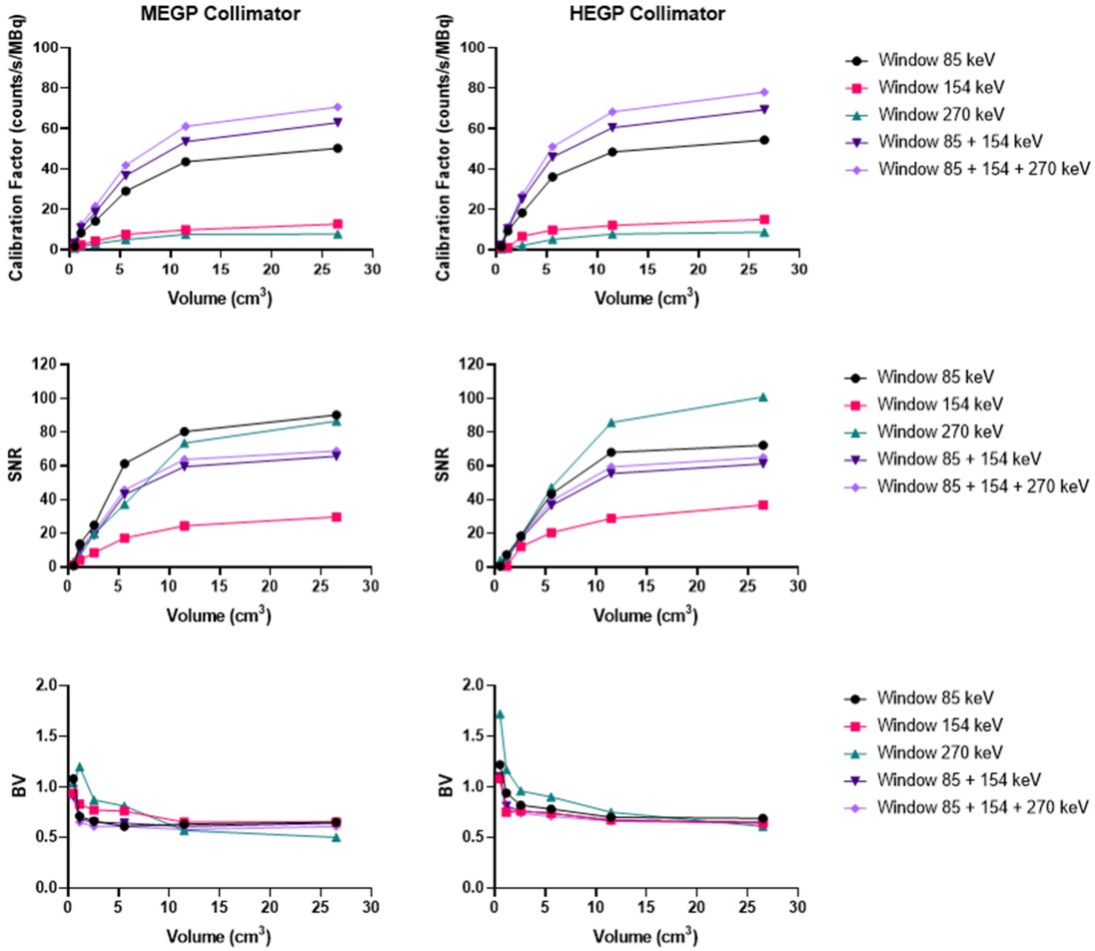
Calibration factors, SNR and BV measured on SPECT/CT images of the NEMA phantom filled with ^223^Ra for each energy window and combination of the energy windows. Representative data acquired on the Discovery 670 (GE) with the MEGP (left column) and HEGP (right column) collimators.

**Figure 9:**
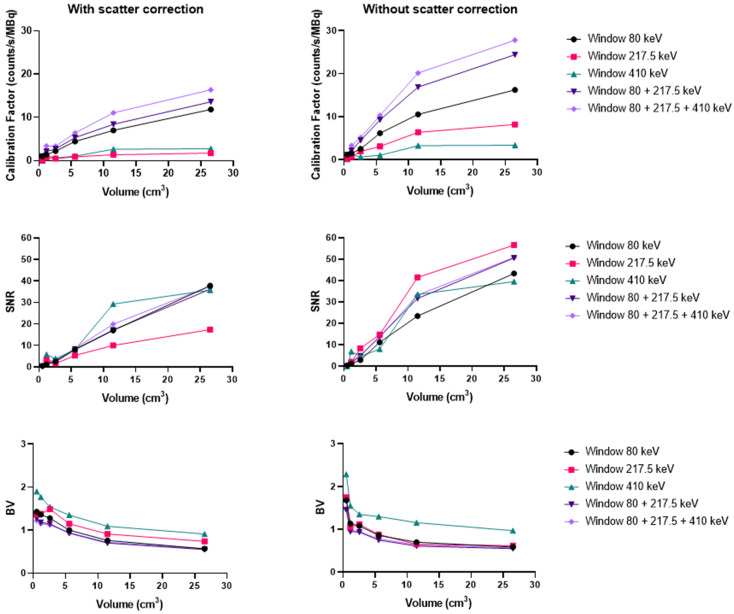
Calibration factors, SNR and BV measured on SPECT/CT images of the NEMA phantom filled with ^225^Ac for each energy window and combination of energy windows. The images were acquired on the Optima 640 (GE) with the HEGP collimator with scatter correction (left column) and without (right column).

**Figure 10:**
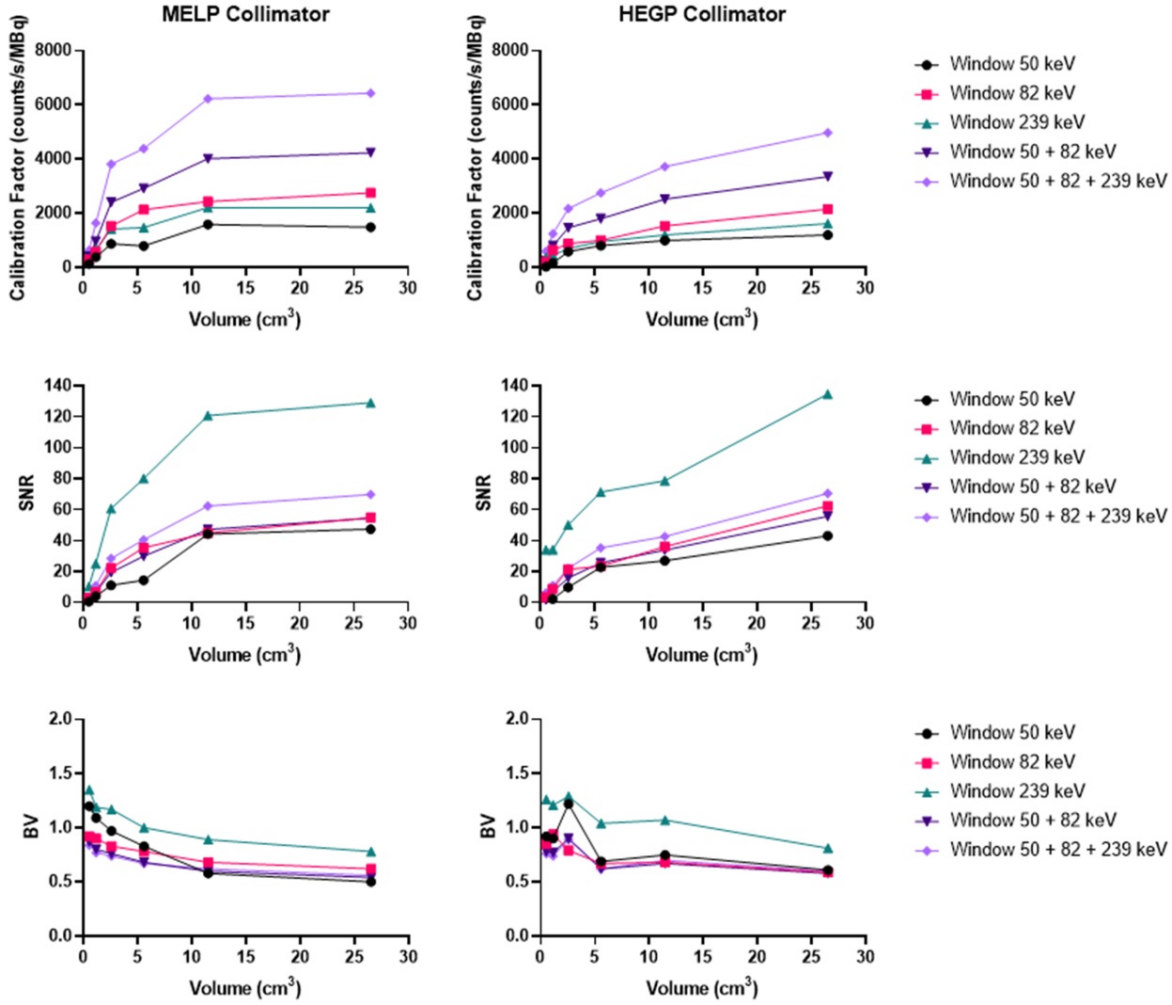
Calibration factors, SNR and BV measured on SPECT/CT images of the NEMA phantom filled with ^227^Th for each energy window and combination of energy windows. The representative data were acquired on the Symbia T6 (Siemens) with the MELP (left column) and HEGP (right column) collimators.

On the Symbia T6, the MELP collimator offered a better calibration factor and SNR: 12.6% and 9.4% for the second largest sphere. However, the BV was higher: 3.2% for the second largest sphere (Figure 6 and Figure 10).

To summarize, the Symbia T6 with the MEGP collimator gave the highest calibration factor and SNR: 49.5% and 10.7% compared to the Discovery 670 and 49.5% and 9.4% compared to the Optima 640 for the largest sphere. Moreover, the BV was less important in the images obtained with the Symbia T6: 6.9% compared to the Discovery 670 and 4.8% compared to the Optima 640 for the largest sphere.

**Figure 11:**
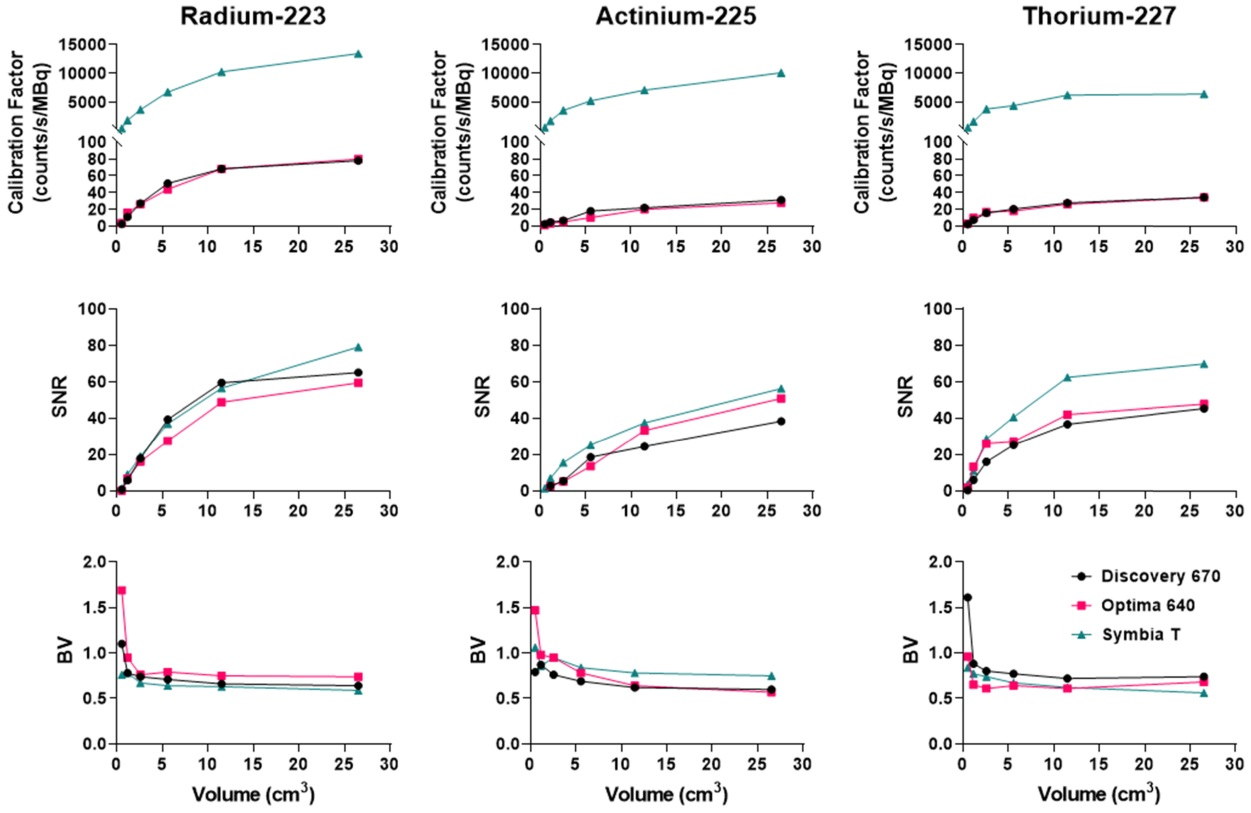
Comparison of the calibration factors, SNR and BV between gamma cameras for the three isotopes. Left: Radium. The NEMA phantom was acquired with the HEGP collimator for the Discovery 670 and the Symbia T6 and with the MEGP collimator for the Optima 640. Center: Actinium. The NEMA phantom was acquired with the HEGP collimator for the Optima 640 and the Symbia T6 and with the MEGP collimator for the Discovery 670. Right: Thorium. The NEMA phantom was imaged with the MEGP/MELP collimator for the three gamma-camera.

Figure 11 recapitulates the calibration factor, SNR and BV measured for the three isotopes on the optimally determined configuration for each system.

### Limit of detection

**Figure 12:**
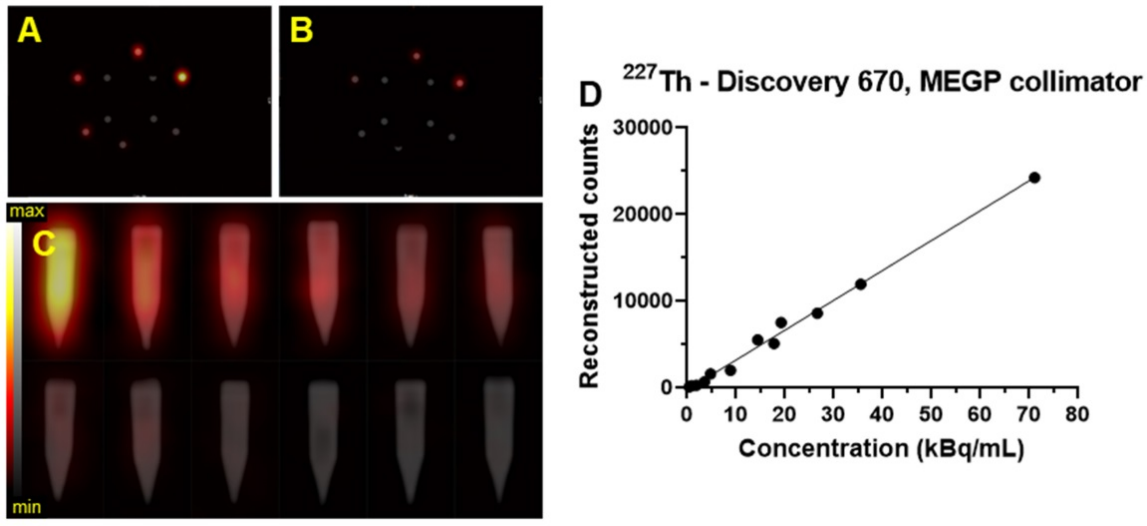
Transaxial and coronal slice from the SPECT/CT fusion of the Limit of Detection phantom acquired on the Discovery 670 (GE) with the MEGP collimator for ^227^Th. A) Limit of detection phantom with higher activities scale of ^227^Th. B) SPECT of lower scale limit of detection phantom. C) Gradient of activities per sample arranged in order of activity concentration (high: 74, 37, 27.7, 18.5, 9.2 and 3.7 kBq/mL, and low: 20, 15, 5, 2, 1 and 0.5 kBq/mL). D) Plot of the number of reconstructed counts detected in the hot tubes as a function of concentration.

Figure 12 shows an example of the SPECT/CT acquisition of the detection limit measurements. For the high activity phantom, all of the sample activities were visible except the lowest activity concentration (3.7 kBq/mL) (Figure 12A-C). Using the phantom with a lower activity concentration range; only the three higher activity concentrations were detectable above background: 20, 15, and 5 kBq/mL (Figure 12B-C).

The detection limits phantoms filled with ^223^Ra and ^227^Th were imaged with the Discovery 670 (GE) equipped with a HEGP collimator and the MEGP collimator, respectively. The detection-limit phantoms filled with ^225^Ac were imaged with the Symbia T6 (Siemens) equipped with a HEGP collimator. The limit of detectability was determined as 5 kBq/mL for every isotope, and the linearity of response was also assessed (Figure 12D).

### Validation with anthropomorphic physical phantoms

**Figure 13:**
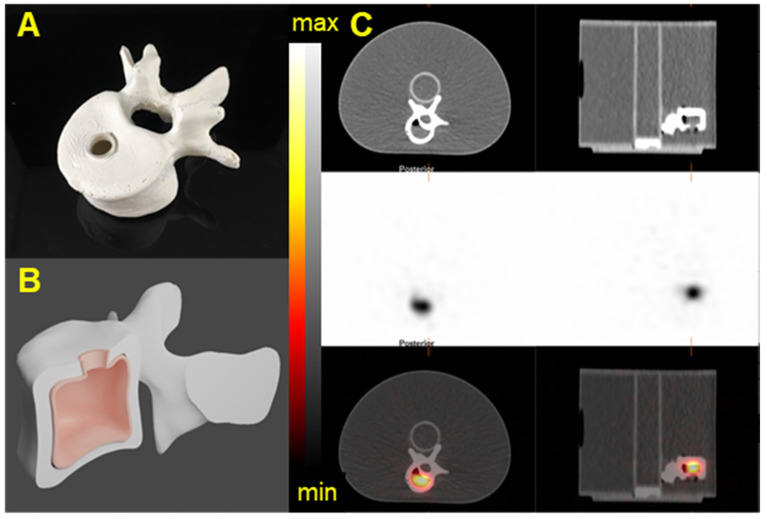
3D-printed Vertebrae phantom. A) Picture of a 3D printed Vertebrae. B) CAD of the Vertebrae showing the fillable space. C) The Vertebrae phantom was fixed on the cylindrical insert inside the NEMA phantom SPECT/CT images of the 3D-printed vertebrae phantom acquired on the Discovery 670 with the MEGP collimator. Transaxial and Sagittal views of the Vertebrae phantom filled with 40 kBq/mL of ^223^Ra. Top: CT view. Middle: NM view. Bottom: NM/CT fusion.

In order to evaluate tomographic image quality in a controlled real-world scenario, we evaluated anthropomorphic 3D-printed structures. Figure 13 and Figure S12 show SPECT/CT acquisitions of the vertebrae and tumor phantoms (described in Methods) filled with ^223^Ra, ^225^Ac, and ^227^Th. Table 6 summarizes quantification of these images obtained by using the calibration factor measured on the NEMA phantom for the largest sphere 36. The results show the possibility to quantifying the activity of ^223^Ra and ^225^Ac with an error lower than 4%. In the case of ^227^Th, quantification was still possible but with a substantially larger error.

## Discussion

This study investigates the capabilities to acquire and quantify SPECT of three ⍺-emitting radioisotopes: ^223^Ra, ^225^Ac, and ^227^Th at activity levels commensurate with RPT applications on clinically relevant imaging systems. Three state-of-the-art gamma cameras that are widely deployed for conventional diagnostic nuclear medicine applications were evaluated: the Discovery 670 and Optima 640 from GE and the Symbia T6 from Siemens, and energy windows and collimator settings were tested for their impact on image quantitation.

Of the α-emitting agents used in imaging studies, parameters for ^223^Ra and ^225^Ac with planar imaging have been the most widely reported 13-15, 18, 19, 21, 22, 28, 42. Quantitative SPECT investigations are much less common, involving both ^223^Ra 23-25 and ^225^Ac 43-46. Most recently, a case report has demonstrated the feasibility of dosimetry on a patient with a PSMA-targeted ^225^Ac agent 30. The authors used HEGP collimation and two (218 keV and 440 keV) energy windows, but quantification of activity concentration was not reported. To date, no peer reviewed study of ^227^Th SPECT/CT has been published, despite the active interest in this area 47 and the potential of scintigraphy for ^227^Th quantification 48.

To comprehensively evaluate ⍺-emitter SPECT, an optimized acquisition protocol was first determined for each isotope. To that end, an energy spectrum was acquired on both the GE Discovery 670 and the Siemens Symbia T6 in order to determine appropriate energy windows. Diverging from the most commonly used tracers (Technetium-99m and Indium-111), the reconstruction protocol was optimized by acquiring SPECT/CT of the NEMA phantom. We used vendor-recommended reconstruction parameters, including the number of iterations and subsets and post-reconstruction filters. These reconstruction parameters varied between systems. The role of scatter correction in activity estimation was investigated, along with limits of detection, for each isotope and each system. Finally, the acquisition and reconstruction protocol were validated using anthropomorphic 3D-printed phantoms.

Our data establish that three-energy emission windows are recommended in order to provide higher count statistics and improve the image quality. While SNR can degrade with a multiwindow approach, the improved sensitivity is important towards the eventual goal of quantification of patient tissue activity concentrations. Scatter correction using the DEW technique is not recommended for ^225^Ac and ^227^Th as it decreases sensitivity. However, scatter correction has the intended effect of decreasing noise in reconstructions. Since these ⍺-emitters have low detectable photons, we suggest that sensitivity is an important study design criterion. More recently, methods that been proposed which use the scatter-window data for the quantification task 49, which may achieve the goals of both improving sensitivity and accomplishing scatter correction.

The NEMA phantom studies allow one to adapt the calibration factor for different lesion sizes 39. The calibration factor determined from our acquisitions decreases with decreasing sphere volume. For smaller spheres, the visual contrast and calibration factor decreased due to partial volume effects. The 3D-printed tissue phantoms showed that quantification is achievable with the three alpha-emitters on the three different systems.

The choice of the collimator is a tradeoff between the sensitivity of the measurement and the observed signal to noise ratio. The characteristics of both collimators used for each system were summarized in Table 3; briefly, the MEGP collimator has smaller hole diameter and thinner and shorter septa. The HEGP collimator prevents more unscattered photons from being detected compared to the MEGP collimator owing to the thicker and taller septum. The thicker septa help reduce the septal penetration by higher energy photons. This explains why the SNR measured in the higher energy window with the HEGP collimator is greater the MEGP, for each isotope.

At our center, for the imaging of ^223^Ra, we recommend using the Discovery 670 with the HEGP collimator. The HEGP and Symbia T6 is recommended for imaging ^225^Ac. Finally, the Symbia T6 with the MEGP collimator is suggested for imaging ^227^Th. These configurations are derived from a compromise between sensitivity and noise characteristics, illustrated in Figure 11. Overall, our findings also demonstrate that the Symbia T6 is more sensitive under the test conditions. This result might be explained by the larger crystal dimensions, as well as differences in signal processing electronics. The calibration factor measured on the Symbia T6 is 50% higher than the one assessed on both GE cameras for each sphere and isotope evaluated.

We have observed that under our experimental SPECT conditions, system configuration and image quality with ^223^Ra was greater than for ^225^Ac and ^227^Th. This is likely due to the fact that the gamma emission abundance for ^223^Ra exceeds that of the other isotopes and its photopeaks coincide with a range (100-300 keV) for which detector efficiency is highest. In addition, higher energy gamma rays, as observed with ^225^Ac, cause performance degrading effects such as increasing septal penetration and down-scatter that complicate reconstruction and quantification tasks.

Our measurements under the various experimental conditions in this work have import for interpretation and design of clinical SPECT/CT efforts. In clinical practice, patients receive 55 kBq/kg of ^223^Ra every 4 weeks for 6 cycles. Pharmacokinetic studies have shown that the principal organ at risk is the bone marrow 7, 17. In Pacilio *et al.* 22 and Murray *et al.* 15, the authors reported an average total lesion size of 87 mL (1.2 to 270 mL in 14 patients and 53 lesions) for osteoblastic bone metastasis of prostate cancer. However, in the context of osteolytic bone metastasis of kidney cancer, the target of new clinical trials 50, lesions are much smaller (average volume of 0.6 mL specifically, 0.1-5.1 mL in 10 patients and 66 lesions) 51. Therefore, taking into consideration a limit of detection of 5 kBq/mL, the lowest detectable activity in an averaged sizes osteoblastic lesion would represent approximately 10% of the injected activity for an 80 kg patient. In the case of an osteolytic lesion, it would represent about 0.07% of the injected activity for the same patient. Thus, we predict that our protocol will enable the quantification of osteoblastic bone metastasis (with a significant uptake) whereas, for osteolytic bone metastasis, partial volume correction will be necessary to obtain a more robust quantification.

A range of peptide and antibody ^225^Ac-formulated agents are under clinical trial evaluation. ^225^Ac-PSMA-617 is perhaps the most well described 26-28. Consequently, lesions dimensions are similar as described above for ^223^Ra. Our study shows that the quantification of ^225^Ac SPECT images is achievable. Recently, Gosewisch *et al.* demonstrates feasibility of quantitative SPECT imaging in a patient injected with ^225^Ac-PSMA 30. The authors used two energy windows centered on the 218 and 440 keV photopeaks. They were able to perform a dosimetry study on the patient kidneys and a small lesion. Renal clearance of low molecular weight radiopharmaceuticals makes the kidney an organ of considerable interest for these efforts. The use of a specific phantom such as a 3D-printed kidney phantom 52 may be necessary to properly calibrate a gamma camera for the specific task of quantitative alpha particle emitting agent SPECT. Indeed, studies have shown that spill-out effects become more relevant in organs whose shapes differ significantly from a spherical geometry and that sphere-based calibration factor lookup tables should be replaced by a more geometry-specific alternative 53, 54. Moreover, VOI delineation for nonuniform structures is a significant contributor to uncertainty in RPT dosimetry 55, and it is important to optimize the calibration for ill-defined objects. Similarly, SPECT methods that quantify uptake directly from the projection data show promise for improved quantification even with non-spherical structures evaluated for ^223^Ra 56, and this approach may be expanded to include other emitters.

Phase I clinical trials using ^227^Th conjugates targeting lymphoma, mesothelioma and prostate cancer are ongoing (NCT02581878, NCT03507452, NCT03724747). For ^227^Th-labeled anti-PSMA agents, as for ^223^Ra and ^225^Ac-labeled PSMA-inhibitors, our protocol demonstrates the feasibility of quantifying lesion uptake. Previous work by Hammer *et al.* 57 has identified the organs at risk as the liver and spleen. Similar to ^225^Ac, the use of organ specific phantoms may be necessary for accurate calibration of the gamma cameras.

The initial daughter of ^227^Th is ^223^Ra, which may have an independent biodistribution to that of the parent conjugate. Quantitative *in vivo* imaging with conventional systems is challenging due to cross-talk between neighboring gammas as well as scattered gamma photons. This might explain a greater difference in the quantification results from our realistic phantoms studies. In the present study, all the ^227^Th acquisitions have been performed within 24 h after purification, and further study may be required to evaluate quantification at later timepoints from in-growth of daughter emitters. Larsson *et al.* 48 and Murray *et al.* 34 have proposed a methodology using different energy windows for planar imaging in this context. Li *et al.* 56 proposed a multiple-energy-window projection-domain quantification method that jointly estimates the regional activity uptake of both ^227^Th and ^223^Ra directly using the SPECT projection data from multiple energy windows. This method yielded accurate quantification and further work to validate planar and SPECT efforts are ongoing.

A primary focus in this work was hardware configuration evaluation of multiple isotopes, however software considerations in the clinical setting are also important. The acquisition protocol for each isotope has been determined and optimized on each systems' software, using settings recommended by the vendor. Nevertheless, the reconstruction and post-reconstruction settings used in this study could be further optimized for quantification. Indeed, precise reconstruction methods have to take into account the scatter and attenuation of each isotope emission in the system matrix of the iterative reconstruction algorithms. In our experience, clinical software workflows may not at this time be well suited for multiple photon emissions. Thus, more advanced methods, such as those based on Monte Carlo simulations, may help improve the accuracy of SPECT/CT reconstruction and thus quantify uptake more accurately.

## Conclusion

SPECT/CT imaging is, and will continue to be, an important tool in the development of new therapeutic radiopharmaceuticals, as it provides a means to assess the biodistribution in patients following administration. However, great care has to be taking for system calibration and acquisition protocol development and implementation. The present work demonstrates quantification of ^223^Ra, ^225^Ac, and ^227^Th activity on SPECT/CT images is feasible on standard double-headed SPECT/CT which are widely deployed in the field. Further, configurations have been elaborated that will enable accurate detection and quantitation of activity concentrations from α-emitters in clinical use. The methodology developed in this study will be useful for the other α emitting isotope of clinical interest such as ^212^Pb or ^211^At.

## Supplementary Material

Supplementary figures.

## Figures and Tables

**Table 1:** Decay data for ^223^Ra, ^225^Ac and ^227^Th and their daughters. The bolded energies are within the ideal energy range of the gamma camera

Isotope	Half-life (days)	Daughters				Principal emitted photon energies (keV) (% abundance)
^223^Ra	11.43					11.7 (25.0), **81.1** (15.0), **83.8** (24.9), **94.9** (11.3), 144.2 (3.2), **154.2** (5.6), **269.5** (13.7) and 323.9 (3.9)
		^219^Rn				**271.2** (10.8) and 401.8 (6.4)
			^211^Pb			404.9 (3.8) and 832.0 (3.5)
				^211^Bi		**351.1** (12.9)
^225^Ac	10					12.0 (6.9) and 14.8 (6.7)
		^221^Fr				**218.2** (11.6)
				^213^Bi		**440.5** (26.1)
^227^Th	18.68					12.3 (21.0), 50.1 (8.0), **236.0** (12.3) and 256.3 (7.0)
		^223^Ra				11.7 (25.0), **81.1** (15.0), **83.8** (24.9), **94.9** (11.3), 144.2 (3.2), **154.2** (5.6), **269.5** (13.7) and 323.9 (3.9)
			^219^Rn			**271.2** (10.8) and 401.8 (6.4)
				^211^Pb		404.9 (3.8) and 832.0 (3.5)
					^211^Bi	**351.1** (12.9)

**Table 2:**
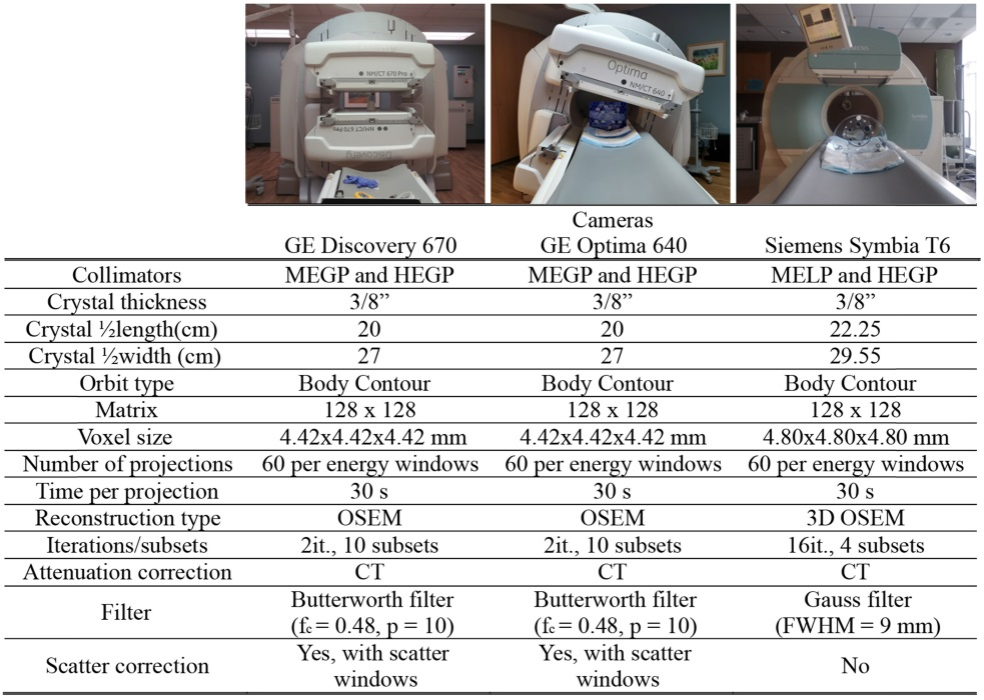
SPECT/CT systems specification and setup for ⍺-SPECT imaging

**Table 3:** Collimator Characteristics

SPECT/CT Camera	Collimators	Hole diameter (mm)	Septal Thickness (mm)	Hole Length (mm)
GE 670 and 640	MEGP	3	1.05	58
	HEGP	4	1.8	66
Siemens Symbia T6	MELP	2.94	1.14	40.64
	HEGP	3.4	2	50.8

**Table 4:** Emission and scatter energy windows chosen for each radioisotope on the Discovery 670 (GE)

Windows	^223^Ra		^225^Ac		^227^Th	
	Emission windows	Scatter windows	Emission windows	Scatter windows	Emission windows	Scatter windows
I	85 keV ± 20%	57 keV ± 17,5%	80 keV ± 20%	57 keV ± 10.7%	50 keV ± 17.5%	36 keV ± 13.8%
II	154 keV ± 20%	113 keV ± 8.8%	217.5 keV ± 8%	165 keV ± 21.2%	82 keV ± 19%	62.5 keV ± 5.6%
III	270 keV ± 10%	226.5 keV ± 7,2%	410 keV ± 6.1%	365.5 keV ± 4.8%	239 keV ± 9%	200 keV ± 5%

**Table 5:** Emission and scatter energy windows chosen for each radioisotope on the Symbia T6 (Siemens)

Windows	^223^Ra	^225^Ac	^227^Th
I	85 keV ± 20%	82 keV ± 20%	50.6 keV ± 20%
II	154 keV ± 20%	216.8 keV ± 8%	85.7 keV ± 15%
III	270 keV ± 10%	444.3 keV ± 5%	240 keV ± 9%

**Table 6:** Summary of quantification results with the 3 isotopes measured on calibrated SPECT/CT images acquired on the Discovery 670 with the MEGP collimator

Isotope	Real activity (MBq)	Measured activity (MBq)	Error (%)
^223^Ra	0.597	0.609	2.1
^225^Ac	0.401	0.386	3.5
^227^Th	0.404	0.356	11.8
