# Supplementary material for: Practical considerations for quantitative clinical SPECT/CT imaging of alpha particle emitting radioisotopes
Source: Theranostics. 2021 Sep 27;11(20):9721–37. doi: 10.7150/thno.63860 (PMC8581409; doi:10.7150/thno.63860)
Supplement: Supplementary file 1 — Supplementary figures. [file thnov11p9721s1.pdf]

Supplemental Data

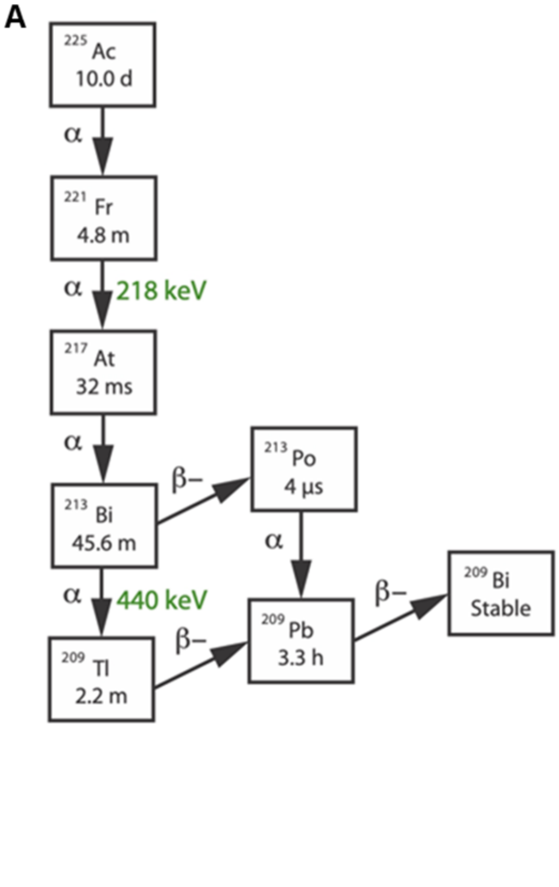

Figure S1 - Decay schemes of ^{225}Ac (A) and ^{227}Th (B) with the principal photon emission.

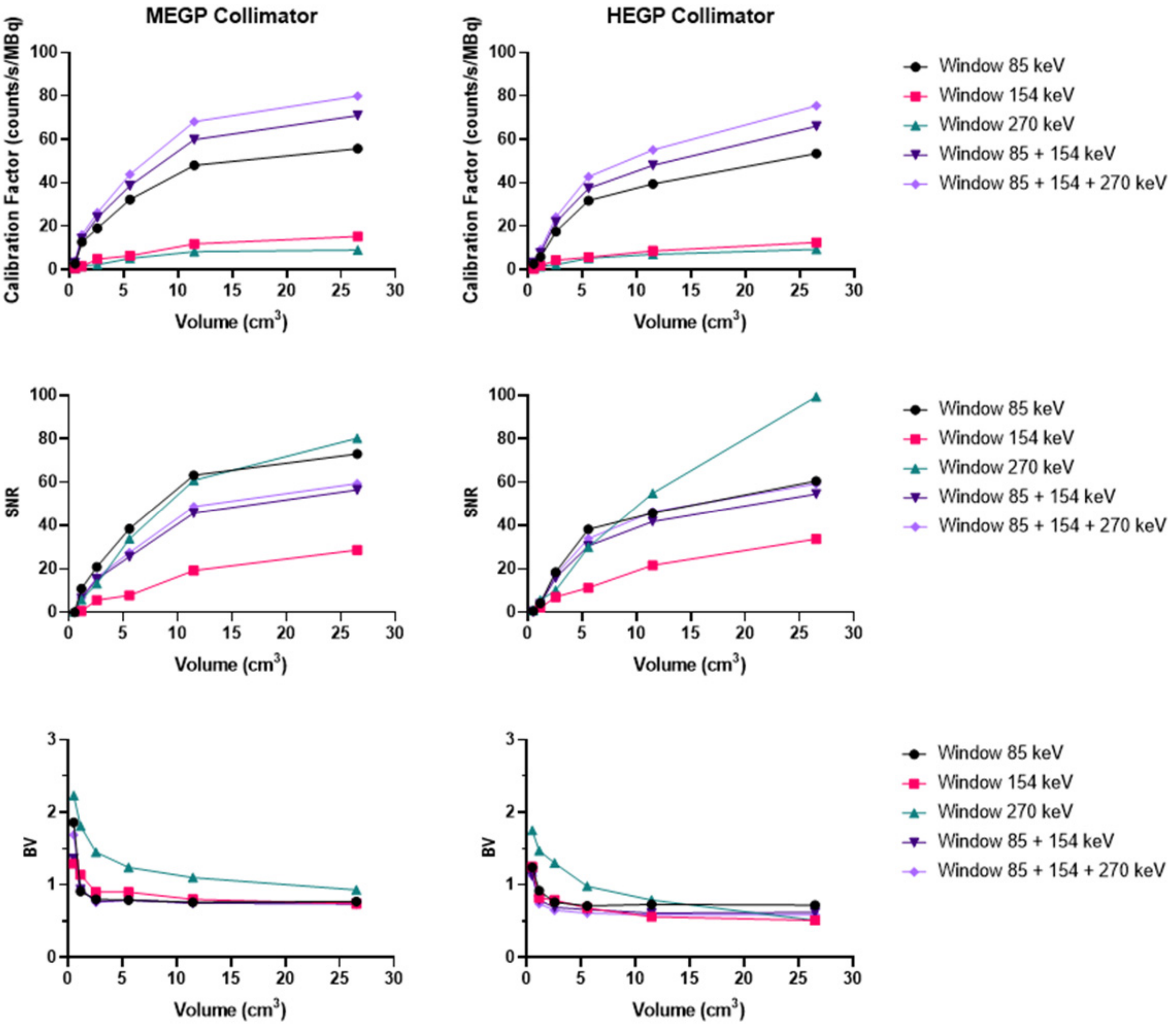

Figure S2 - Calibration factors, SNR and BV measured on the SPECT/CT images of the NEMA phantom filled with ^{223}Ra for each energy window and combination of the energy windows. The images were acquired on the Optima 640 (GE) with the MEGP (left column) and HEGP (right column) collimators.

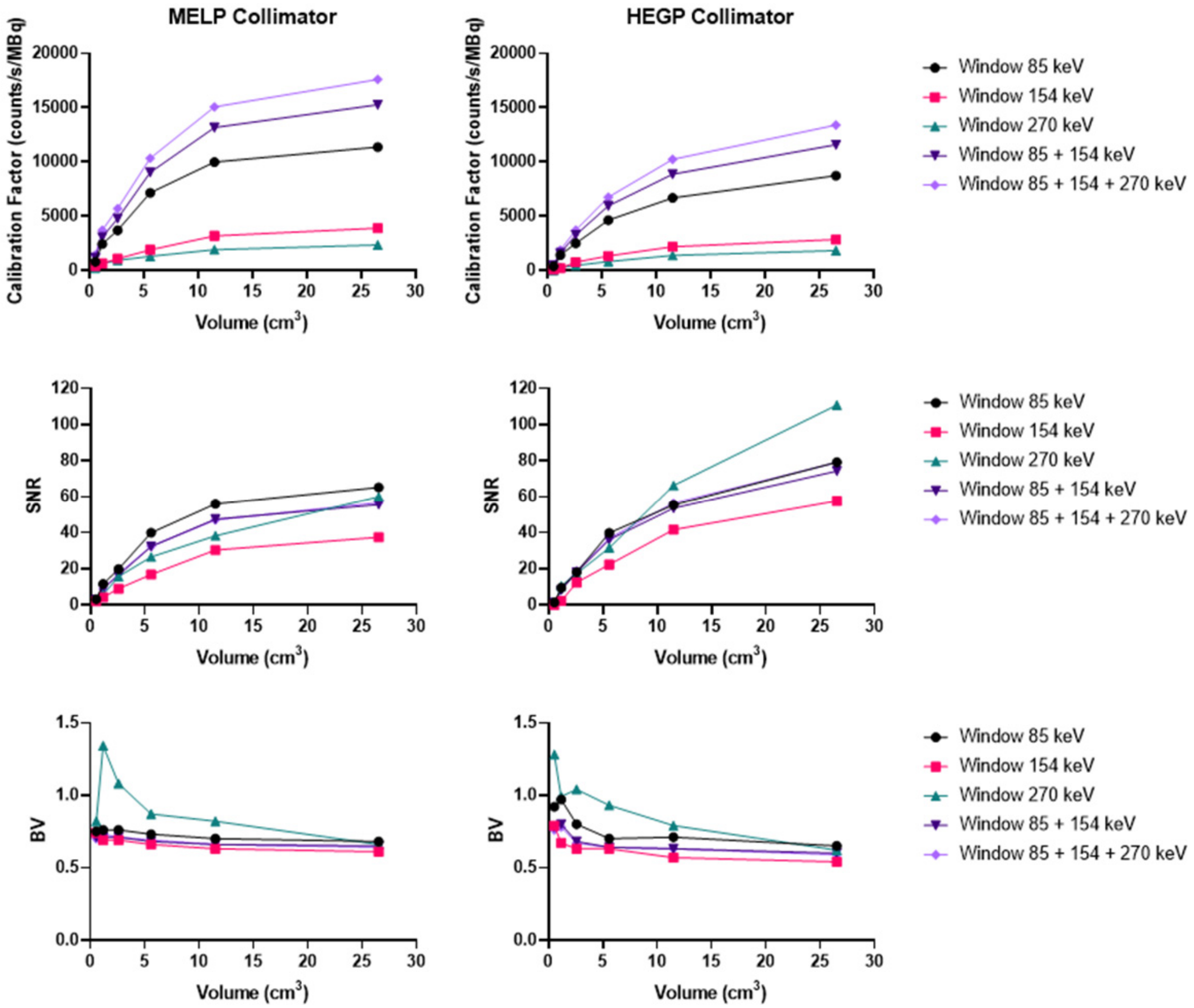

Figure S3 - Calibration factors, SNR and BV measured on the SPECT/CT images of the NEMA phantom filled with ^{223}Ra for each energy window and combination of the energy windows. The images were acquired on the Symbia T (Siemens) with the MELP (left column) and HEGP (right column) collimators.

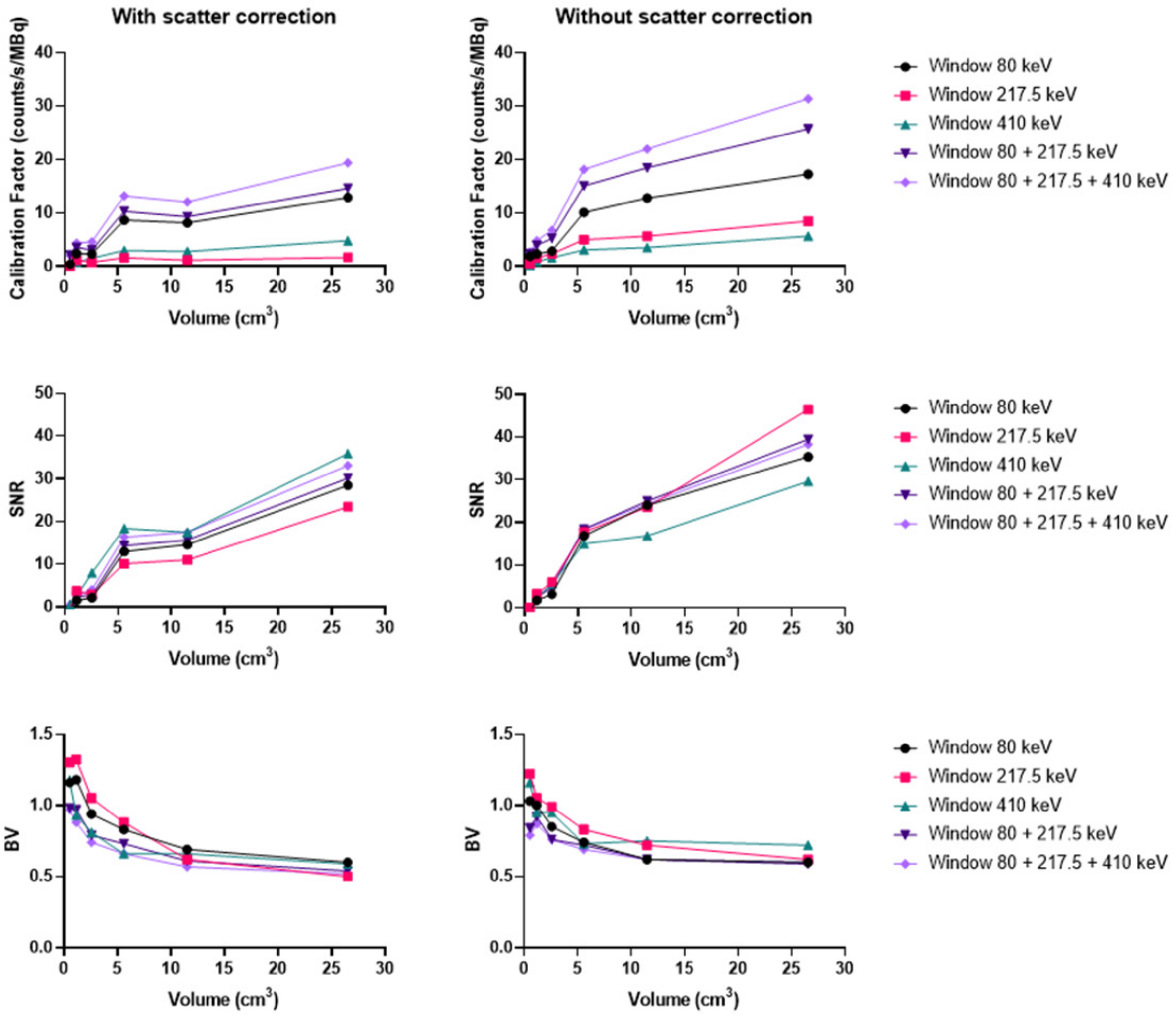

Figure S4 - Calibration factors, SNR and BV measured on the SPECT/CT images of the NEMA phantom filled with ^{225}Ac for each energy window and combination of the energy windows. The images were acquired on the Discovery 670 (GE) with the MEGP collimator with scatter correction (left column) and without (right column).

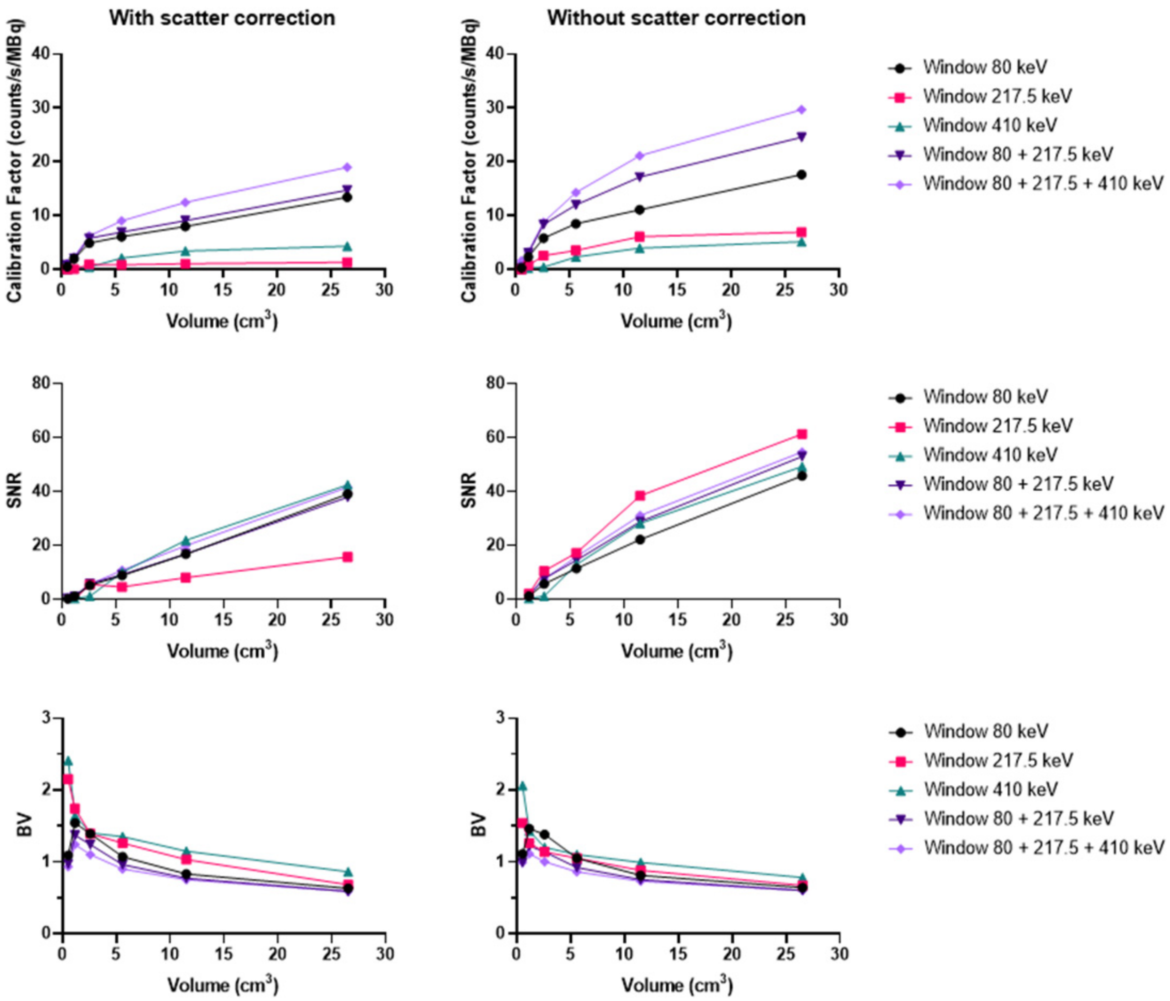

Figure S5 - Calibration factors, SNR and BV measured on the SPECT/CT images of the NEMA phantom filled with ^{225}Ac for each energy window and combination of the energy windows. The images were acquired on the Discovery 670 (GE) with the HEGP collimator with scatter correction (left column) and without (right column).

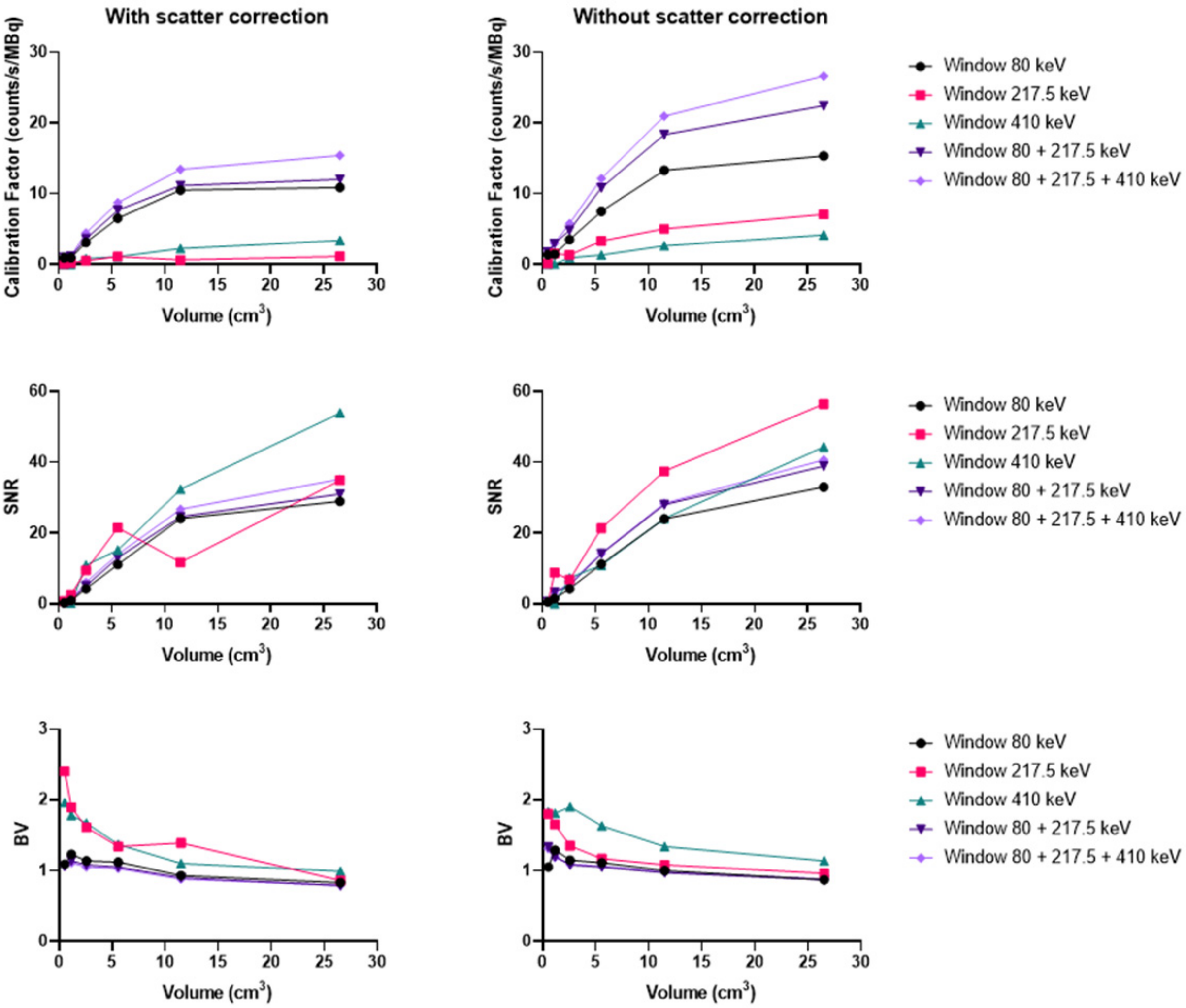

Figure S6 – Calibration factors, SNR and BV measured on the SPECT/CT images of the NEMA phantom filled with ^{225}Ac for each energy window and combination of the energy windows. The images were acquired on the Optima 640 (GE) with the MEGP collimator with scatter correction (left column) and without (right column).

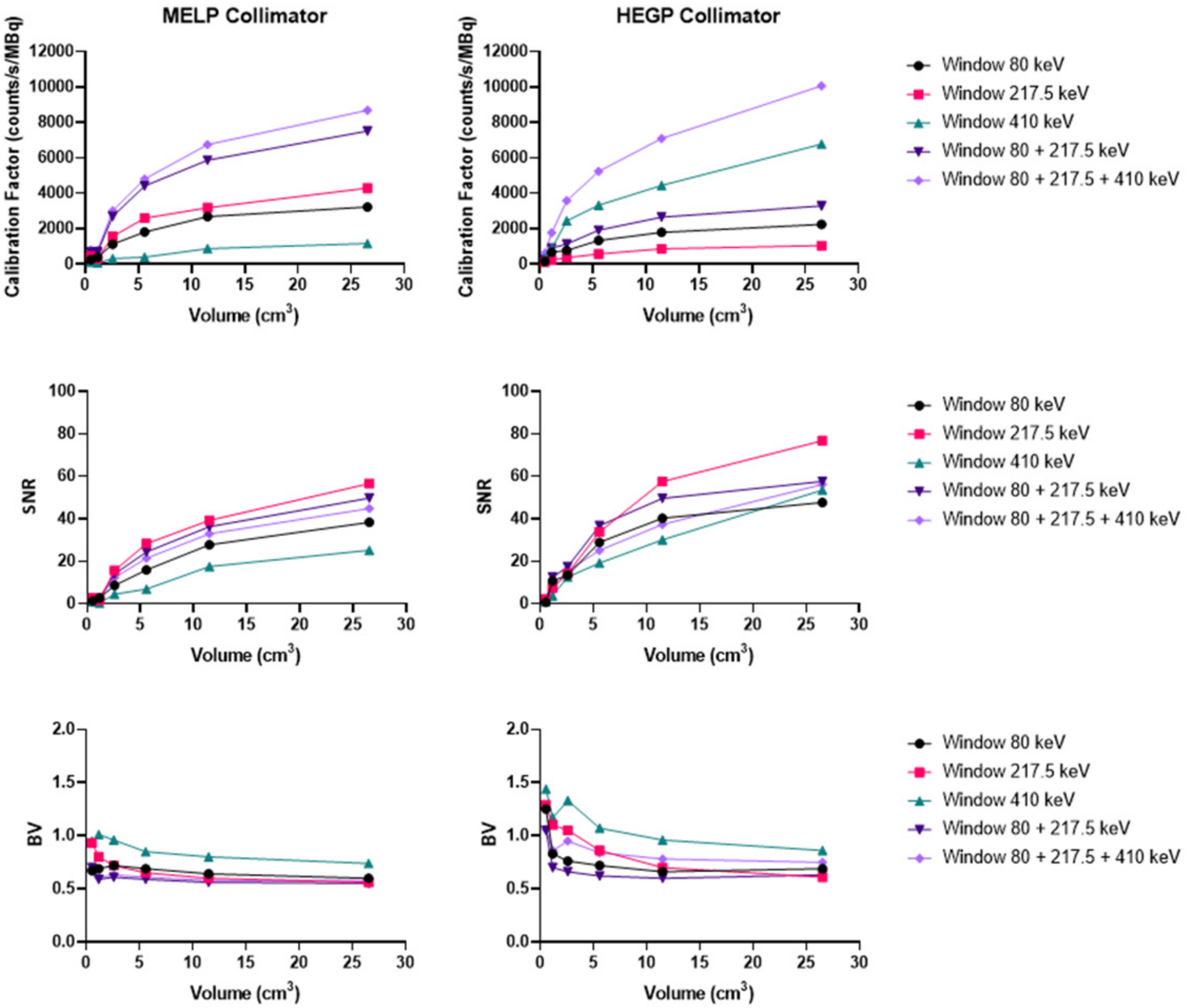

Figure S7 - Calibration factors, SNR and BV measured on the SPECT/CT images of the NEMA phantom filled with ^{225}Ac for each energy window and combination of the energy windows. The images were acquired on the Symbia T (Siemens) with the MELP (left column) and HEGP (right column) collimator.

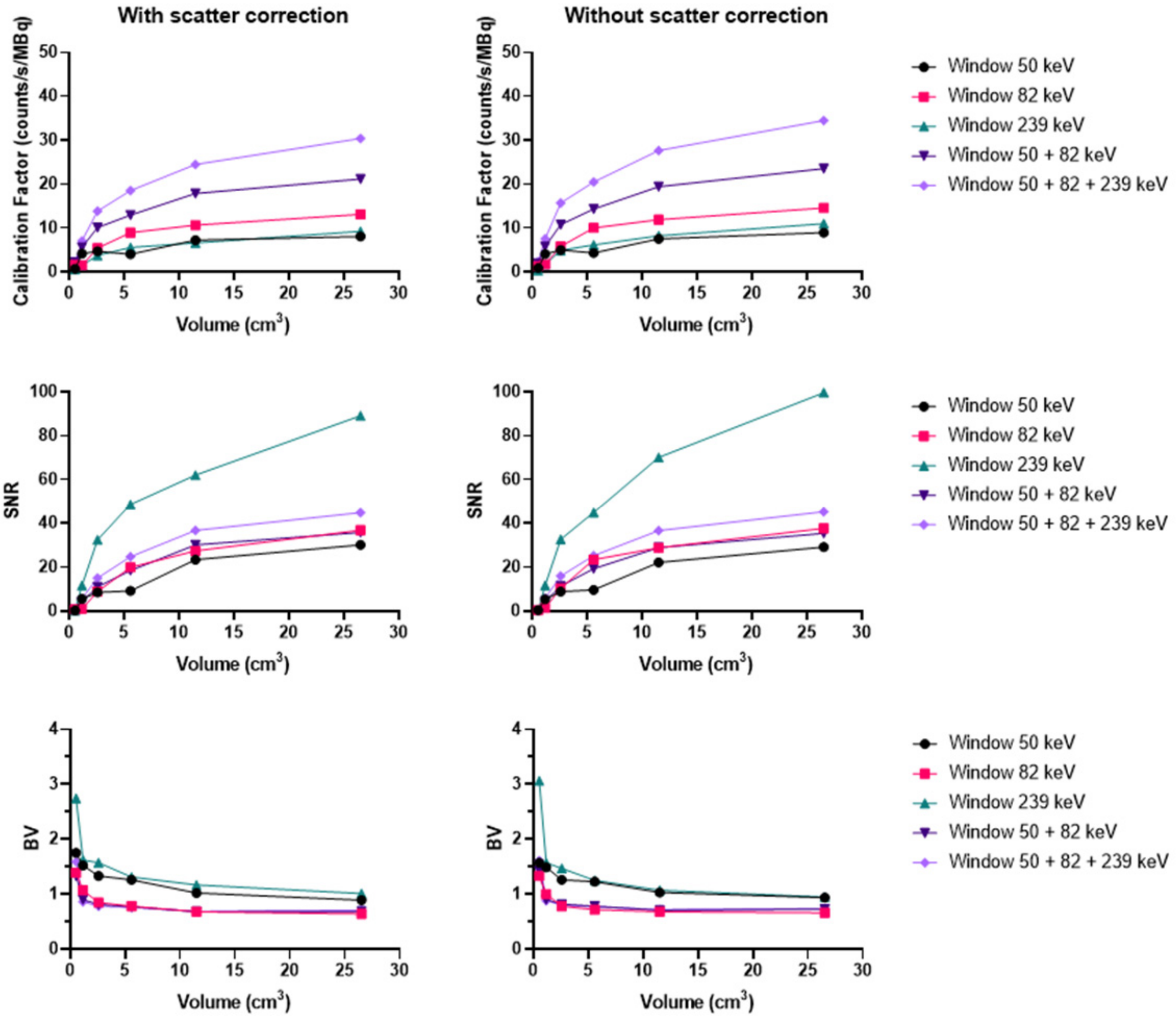

Figure S8 - Calibration factors, SNR and BV measured on the SPECT/CT images of the NEMA phantom filled with ^{227}Th for each energy window and combination of the energy windows. The images were acquired on the Discovery 670 (GE) with the MEGP collimator with scatter correction (left column) and without (right column).

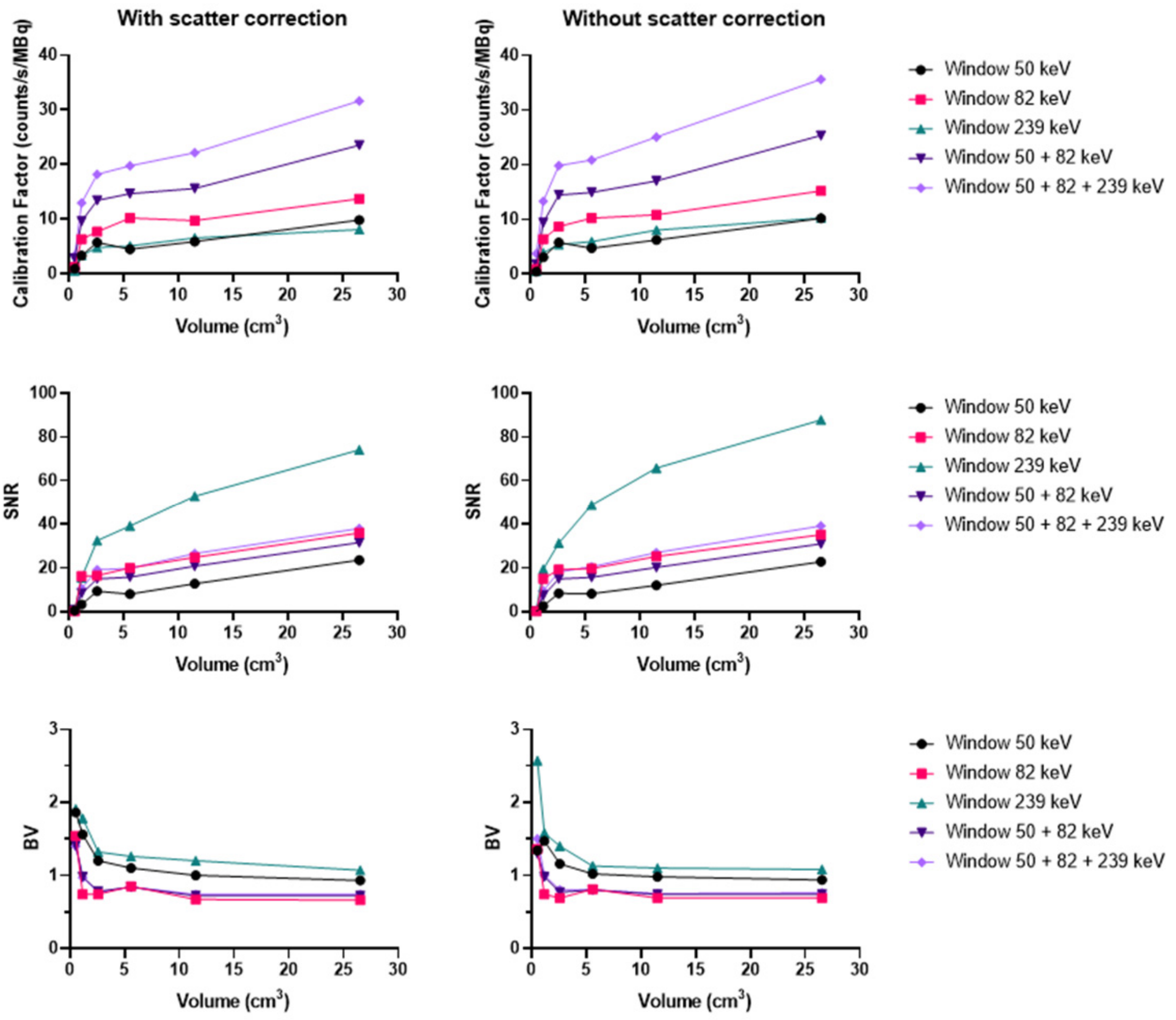

Figure S9 - Calibration factors, SNR and BV measured on the SPECT/CT images of the NEMA phantom filled with ^{227}Th for each energy window and combination of the energy windows. The images were acquired on the Discovery 670 (GE) with the HEGP collimator with scatter correction (left column) and without (right column).

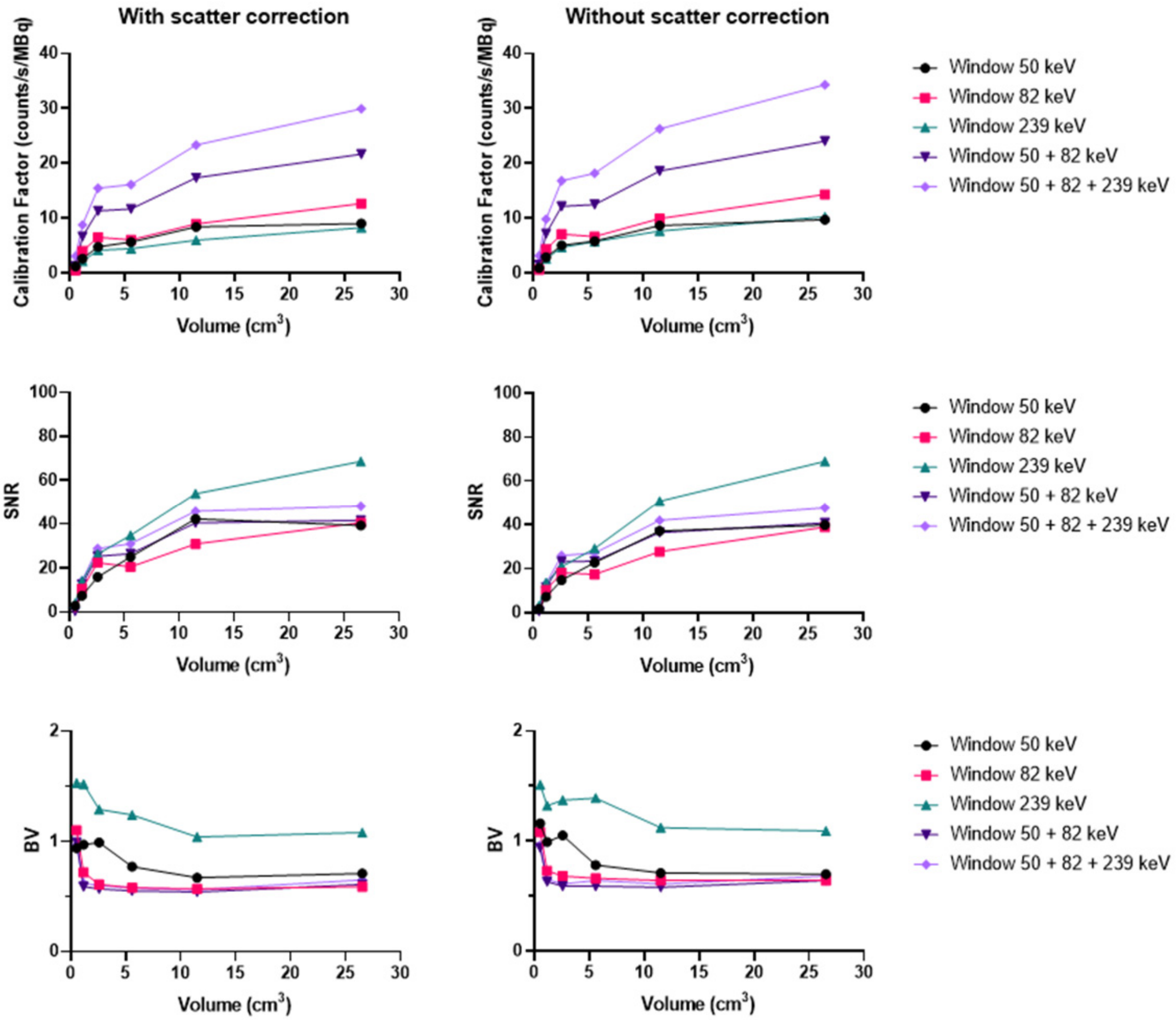

Figure S10 – Calibration factors, SNR and BV measured on the SPECT/CT images of the NEMA phantom filled with ^{227}Th for each energy window and combination of the energy windows. The images were acquired on the Optima 640 (GE) with the MEGP collimator with scatter correction (left column) and without (right column).

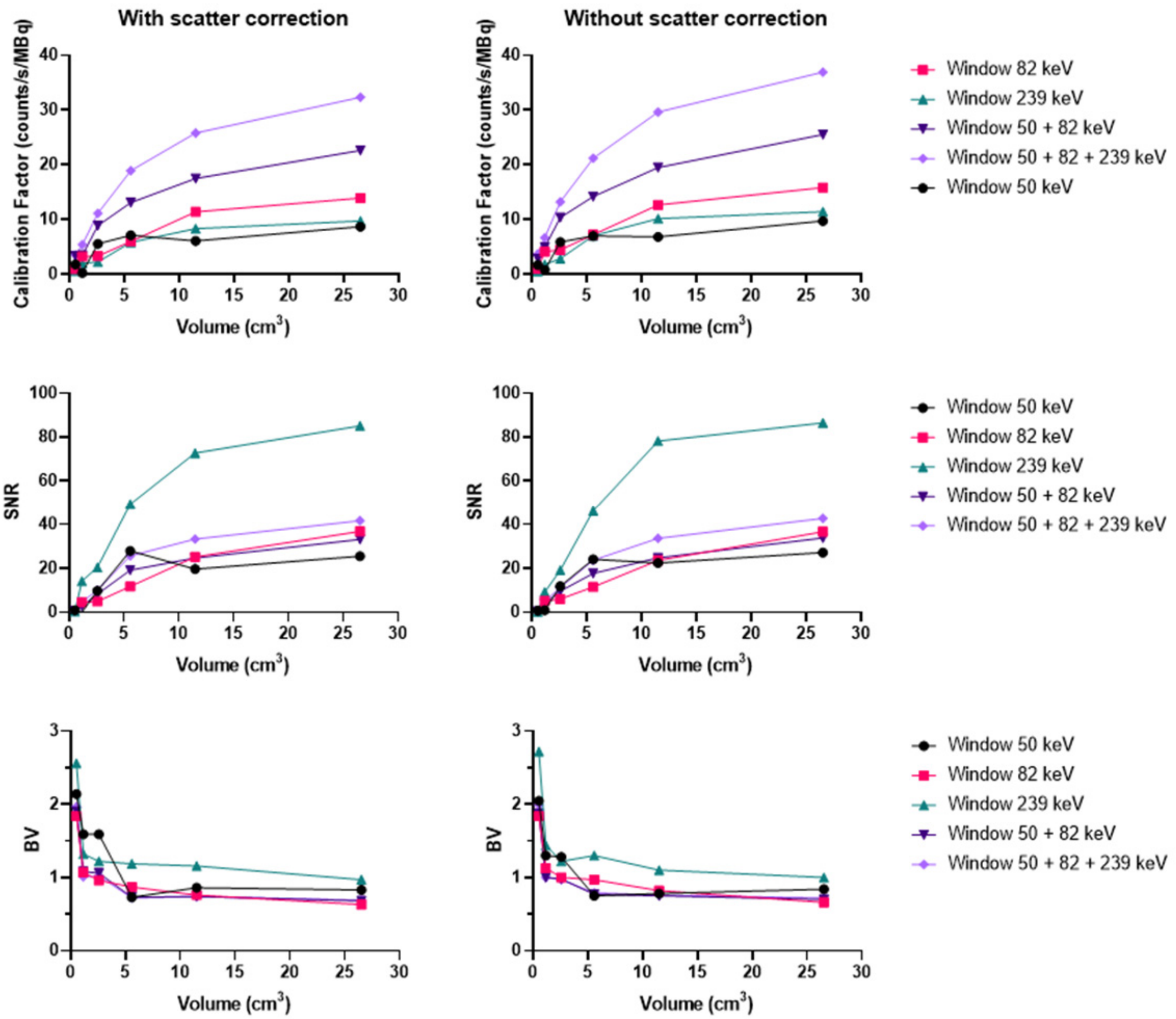

Figure S11 - Calibration factors, SNR and BV measured on the SPECT/CT images of the NEMA phantom filled with ^{227}Th for each energy window and combination of the energy windows. The images were acquired on the Optima 640 (GE) with the HEGP collimator with scatter correction (left column) and without (right column).

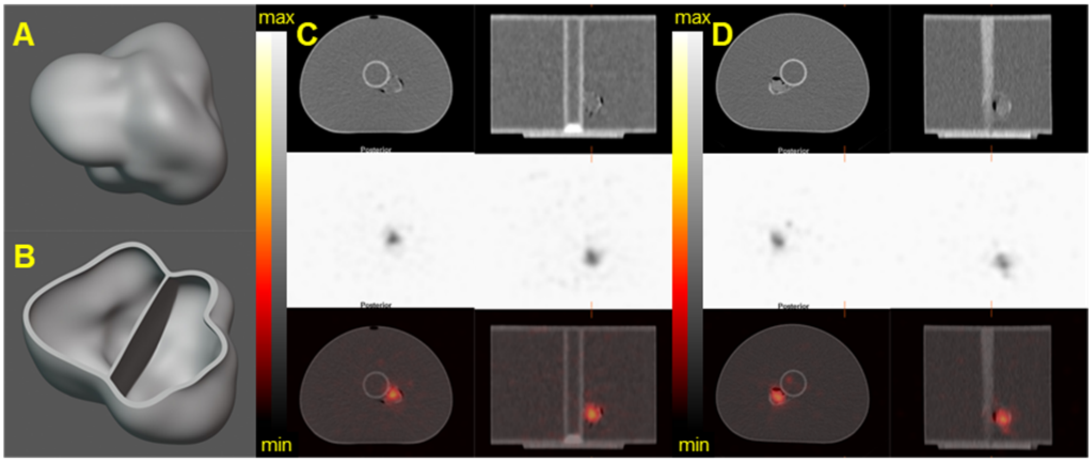

Figure S12 - 3D-printed Tumor phantom. A) and B) CAD model of the tumor and view on the two fillable spaces of the Tumor phantom. The tumor phantom was fixed on the cylindrical insert inside the NEMA phantom. C) and D) SPECT/CT images of the 3D-printed tumor phantom acquired on the Discovery 670 with the MEGP collimator. Transaxial and Transaxial and Coronal views of the Tumor phantom filled with 35 kBq/mL of ^{225}Ac and with 35 kBq/mL of ^{227}Th, respectively. Top: CT view. Middle: NM view. Bottom: NM/CT fusion.
